# Optimization of thermal conductivity in coir fibre-reinforced PVC composites using advanced computational techniques

**DOI:** 10.1038/s41598-025-01471-8

**Published:** 2025-05-14

**Authors:** Saksham Anand, Venkatachalam Gopalan, Shenbaga Velu Pitchumani

**Affiliations:** 1 School of Computer Science and Engineering, Vellore Institute of Technology, Chennai, Tamilnadu 600127 India; 2 Centre for Advanced Materials and Innovative Technologies, Vellore Institute of Technology, Chennai, Tamilnadu 600127 India

**Keywords:** Coir fibre, PVC composites, Thermal conductivity, Optimization algorithms, Response surface methodology (RSM), Particle swarm optimization (PSO), Dragonfly optimization (DFO), Cuckoo search algorithm (CSA), Biodegradable materials, Sustainable composites, Materials science, Mechanical engineering

## Abstract

This research focuses on enhancing the thermal conductivity of coir fibre-reinforced polyvinyl chloride (PVC) composites using advanced optimization techniques. While coir fibre adds sustainability and biodegradability, it poses challenges in achieving optimal thermal performance when integrated into PVC. To address these challenges, the study uses Response Surface Methodology (RSM) and three nature-inspired optimization methods viz. Particle Swarm Optimization (PSO), Dragonfly Optimization (DFO) and Cuckoo Search Algorithm (CSA) to improve factors like fibre content, particle size and chemical treatment. A Box-Behnken experimental design helps to create composite samples using hydraulic injection moulding and thermal conductivity is measured with a two-slab guarded hot plate device. Among the optimization methods, CSA emerges as the most effective, achieving a maximum thermal conductivity of 0.801 W/mK with minimal error deviation (0.01–5.5%) by the process parameters such as potassium hydroxide treatment, coir content of 2 wt% and powder diameter of 75 (µm). DFO delivers consistent results with slightly higher error rates, while PSO demonstrates rapid convergence but greater variability. The comparison shows that CSA performs better, providing a dependable and long-lasting way to create high-quality coir-reinforced PVC composites that are good for industrial use. This work is among the first to compare multiple bio-inspired optimization algorithms for enhancing the thermal properties of coir-reinforced PVC composites, offering a new pathway for developing high-performance, eco-friendly materials for industrial applications.

**Supplementary Information:**

The online version contains supplementary material available at 10.1038/s41598-025-01471-8.

## Introduction

Despite the growing demand for sustainable and high-performance composite materials, optimizing the thermal conductivity of natural fiber-reinforced polymer composites particularly coir fiber-reinforced PVC composites remains a significant challenge due to the complex interactions between material constituents and processing variables. Conventional optimization techniques often fall short in capturing these nonlinearities, leading to suboptimal material performance. Addressing this issue requires advanced computational strategies that can accurately model and refine process parameters to enhance thermal behaviour while maintaining the environmental benefits of natural fibers.

Environmentally conscious materials, that function well, are becoming more and more in demand as people realize the importance of protecting the environment. Natural fibre-reinforced composites have attracted significant interest due to their possibility to replace synthetic materials in a range of technical applications. Out of all of them, coir fibre stands out as a potentially beneficial reinforcing material since it is readily available, reasonably priced, biodegradable and has acceptable mechanical qualities. Coir fibre may greatly improve the qualities of composites, which makes them appropriate for usage in sectors including packaging, construction and automotive where balancing performance and environmental impact is essential^1,2^.

Polyvinyl chloride (PVC) is a thermoplastic polymer that is commonly used in composite materials due to its versatility, durability and resistance to environmental deterioration. PVC is reinforced with natural fibres like coir, which enhances both its mechanical and thermal stabilities thereby making it suitable for use in more demanding applications. Coir fibre (CF) is used in PVC composites to increase toughness, thermal insulation and sound absorption while also reducing the environmental effect of employing synthetic yarns like carbon and glass^3,4^. Even though CFRC mechanical properties have been extensively studied, there is growing interest in understanding more about their thermal behaviour, particularly for applications linked to thermal management in the construction and electronics industries^5,6^.

There are several ways to test thermal conductivity based on thermal characteristics and medium of temperature. There are two basic methods of measurement: When a material is to be examined in complete equilibrium, thus measurements are performed using the steady-state technique. The disadvantage is that the process would take a long time to obtain the required equilibrium. The non-steady state techniques are accomplished by the method of heating up. The benefit is that measurements can be made relatively rapidly. The thermal properties of a composite material are important when deciding whether to employ it in an application where temperature changes are significant. Thermal conductivity, for instance, is a crucial component of materials used for heat exchangers, insulation and other thermal management applications^7,8^. Yang et al.^9^ suggested that the guarded hot plate (GHP) technique is the main way for measuring low to medium thermal conductivity materials. According to Alcocer^10^, the Transient Hot Wire technique is an appropriate method to compute thermal conductivity due to its low-cost construction, accuracy and fast method of measurement. Wilson et al.^11^ conveyed that the laser flash method is employed to identify the thermal behaviours of ceramics and polymers. The key variables, influencing the thermal characteristics of natural fibre composites, are matrix and reinforcement type, incorporation of additives, fibre loading, surface modifications of the fibres and various production methods. Devireddy et al.^12^ investigated the thermal conductivities of jute/banana-based epoxy composites. The longitudinal and transverse thermal properties of the composites were reduced by 34.98% and 44.35% for 10 wt% of banana fibres and 30 wt% jute samples respectively. The specific heat capacity and thermal diffusivity of jute/banana composite were lowered with rising the fibre loading. In Emmanual et al.^13^, the results for the thermal conductivity of Polyvinylchloride ceiling material was 0.17 W/m.K, This falls within the range of 0.023-2.9 Wm^− 1^K^− 1^, which is considered to be good insulating material. Thus, it can be utilized as ceiling material in tropical temperature regions. Kim et al.^14^ investigated the thermal conductivity of thermoplastic polypropylene maleic anhydride-grafted polypropylene (MAPP) board reinforced with hybrid natural fibres (Kenaf, Hemp, Flax, Sisal). Additionally, all the natural fibres were chemically treated with coupling agents (Silane). Authors concluded that factors such as polypropylene (47.5%) / Natural fibre (47.5%) yield high thermal conductivity. Suyuthi et al.^15^ studied that the Liquid smoke (LS) and microwave treatment significantly enhance the tensile strength, thermal stability and surface morphology of Sansevieria trifasciata Laurentii fibers. Using RSM / CCD, optimal conditions (120 min LS immersion, 40 °C microwave for 30 min) improved tensile strength by 37.21%. These findings position treated STL fibers as effective reinforcements for sustainable composites. Iravani et al.^16^ investigated the potential of chemical modification to improve the hydrophobic properties and thermal stability of bamboo fibers and to evaluate the sound absorption performance of raw and modified fibers. The authors observed that chemical modification of natural fibers can lead to the development of more stable and safer porous acoustic absorbers. Minchenkov et al.^17^ focused on the production of new, thermoplastic-based structural pultruded profiles and their application in a PVC (polyvinylchloride) window structure as a reinforcement. The results of the hot box test show that the U-value of the window sash and frame with the composite reinforcement is 12% lower than that of a window with a steel reinforcement. Kirubakaran et al.^18^ focused to understand the effect of different factors, such as the particulate loading and the size (coir and hBN − 1 to 5 wt%; Coir Powder size (100–200 μm) of the particles on composite’s corrosion rates and water absorption properties. The authors observed that SSO algorithm outperforms other algorithms in minimizing both corrosion resistance (CR) and water absorption (WA). The Deng’s Value for SSO reached a maximum of 0.68, while the other algorithms showed comparable but lower performance.

In industrial sectors, where material sustainability and thermal management are crucial, the capacity to customize thermal conductivity in coir fibre-reinforced PVC composites creates enormous opportunities. As coir fibres are naturally insulating, they can increase the thermal resistance of composites, which makes them perfect for use in industries where energy efficiency and environmental friendliness are crucial, such as construction (e.g., wall panels, insulation boards), packaging and the automotive sector. However, maximizing thermal conductivity necessitates a striking balance between several variables, including fibre content, particle size and chemical treatments as they have intricate interactions. This is where metaheuristic algorithms like PSO, DFO and CSA are used in conjunction with sophisticated computational methodologies like Response Surface Methodology (RSM). These tools help to create accurate models and improve many input factors at the same time. Hence researchers can find the best combinations that improve heat performance while keeping the structure strong. Such predictive optimization accelerates material development and reduces experimentation costs, contributing to the design of next-generation bio-composites with customized thermal properties. Ultimately, this approach supports the broader goal of creating high-performance, environmental friendly materials for use in heat-sensitive and energy-efficient applications.

This work aims to examine the thermal conductivity of PVC composites reinforced with coir fibre, with an emphasis on optimizing these properties via sophisticated computational techniques. Response Surface Methodology (RSM) might be quite helpful in this case. It is an arithmetic and accurate technique that is frequently used in both academic and commercial research to optimize processes and enhance product quality. Researchers can determine the optimal conditions for material processing by using RSM to efficiently study the different process parameters interactions and their effect on the intended results^19,20^. This study employs RSM to optimize process parameters like fibre content, particulate size and chemical treatment. These parameters are significant because they improve the coir fibre-reinforced PVC composite’s thermal conductivity^21^.

A key factor in the RSM process’s efficacy is the selection of optimization algorithms. Although helpful, traditional optimization methods frequently cannot handle the complexity of multi-objective optimization issues, which are prevalent in the field of materials science. In this work, sophisticated algorithms like the Cuckoo Search Algorithm (CSA), Dragonfly Optimization (DFO) and Particle Swarm Optimization (PSO) are used to tackle this problem. These algorithms are chosen due to their shown capacity to efficiently traverse the solution space, striking a balance between the processes of exploration (finding new solutions) and exploitation (improving upon already-found solutions) to determine the ideal set of parameters^22,23^. Palanisamy et al.^24^ explored machine learning approaches in natural fibre composites, highlighting the potential of algorithms like Particle Swarm Optimization (PSO) and Dragonfly Optimization (DFO) in predicting and optimizing thermal properties. The study emphasized the role of computational intelligence in tailoring composite materials to meet specific thermal performance requirements. Venkatachalam et al.^25^ employed Response Surface Methodology (RSM)/Box-Behnken Designs (BBD) to analyze the fatigue behaviour of coir fibre-reinforced polyvinyl chloride (PVC) composites. Utilizing ANSYS software for fatigue analysis, the research demonstrated that coir fibres treated with triethoxy(ethyl)silane at a concentration of 6 wt% and a particle size of 75 μm exhibited a maximum fatigue limit of 2.819 MPa. Mahmud et al.^26^ focused on the insulating properties of coir fibre and its performance as a reinforcing material in biocomposite production. The study highlighted coir’s low thermal conductivity, making it suitable for thermal insulation applications in civil engineering. Pitchumani et al.^27^ employed Response Surface Methodology (RSM) integrated with various algorithmic techniques to investigate the mechanical and thermal properties of these composites. The research demonstrated that optimizing parameters such as fiber content and chemical treatment significantly enhance thermal conductivity, underscoring the efficacy of computational approaches in composite material design.

Particle Swarm Optimization (PSO), motivated by the social behaviour of bird flocks and fish schools, is an established optimization method that is effective in continuous problem spaces. PSO has been extensively utilized in recent years for optimizing composite material properties. For instance, Chibueze et al. utilized PSO together with fuzzy logic and the Non-Dominated Sorting Genetic Algorithm II (NSGA-II) to forecast and optimize the mechanical properties of a hybrid composite material for application in golf clubs. Their study proved that the integration of these methodologies considerably enhanced the optimization process concerning the composite’s properties^28^. Similarly, Malghan et al. utilized PSO together with response surface methodology to optimize milling process parameters for aluminium matrix composites. Their study highlighted the efficacy of PSO in determining optimal machining conditions, which yielded improved surface quality and reduced cutting forces^29^. In addition, in composite structure design, PSO has been employed to acquire optimal configurations. A study by Innocente et al. employed PSO to determine the optimal design parameters of fibre-reinforced polymer concrete beams, which yielded cost-effective and structurally optimal solutions^30^.

The Dragonfly Optimization Algorithm (DFO), inspired by the swarming behaviours of dragonflies, has been effectively applied to various complex, multi-modal optimization problems. In the realm of composite materials, Jafari and Chaleshtari utilized DFO for the optimal design of orthotropic infinite plates with quasi-triangular cut-outs, demonstrating its capability to enhance structural load-bearing capacity. Their study highlighted DFO’s effectiveness in navigating complex design spaces to achieve optimal configurations^31^. Furthermore, a comprehensive review by Meraihi et al. discussed various applications of the Dragonfly Algorithm, including its use in engineering design and material optimization. The review underscores DFO’s adaptability and efficiency in handling complex optimization tasks across different domains^32^. In this study, DFO is employed to fine-tune process parameters in composite manufacturing, such as fibre content and chemical treatment levels, to ensure the resulting material to exhibit the desired thermal characteristics. Building upon the methodologies of previous research, this approach leverages DFO’s strengths in exploring complex solution spaces to optimize composite material properties.

The Cuckoo Search Algorithm (CSA), stimulated by the nest parasites of certain cuckoo species, has been effectively applied to various optimization problems, including those in composite material design. For instance, Kaveh et al. utilized CSA for optimal efficiency in reducing the girder’s self-weight while meeting design requirements in the design of multi-span composite box girder bridges. In another study, a hybrid algorithm combining CSA and the Stochastic Paint Optimizer was proposed to optimize truss structures using fibre-reinforced polymer composites under natural frequency constraints. This approach effectively addressed the non-linear and non-convex search spaces characteristic of such optimization problems^33^. Furthermore, a comprehensive review by Yang and Deb discussed the fundamental ideas and applications of CSA, highlighting its efficiency in solving global optimization problems across various domains^34^. In this study, CSA was employed to fine-tune process parameters in composite manufacturing, such as fibre content and chemical treatment levels, to ensure the resulting material exhibits the desired thermal characteristics. Building upon the methodologies of previous research, this approach leverages CSA’s strengths in exploring complex solution spaces to optimize composite material properties.

The integration of RSM with these three state-of-the-art optimization techniques offers a powerful approach to understand and enhance the thermal properties of PVC composites reinforced with coir fibre. This work aims to identify the optimal conditions for composite production by a systematic modification of the process parameters and evaluation of the resulting thermal properties. The ultimate goal was to develop a material that meets the performance requirements of certain applications while also reducing reliance on synthetic materials and utilizing natural fibres to promote environmental sustainability.

The systematic optimization of the thermal characteristics of natural fibre-reinforced composites is lacking in substantial research, particularly for composites that use coir as the reinforcing material. The main goal of earlier research was to maximize mechanical attributes such as wear characteristics, impact resistance and tensile strength^35,36^. While several studies have examined the electric characteristics of natural fibre composites, in attendance are still few that thoroughly examined their thermal properties. Furthermore, compared to more conventional methods that frequently concentrate just on mechanical optimization, the application of RSM in conjunction with PSO, DFO and CSA gives a complex way to optimize these features^37^.

Few researchers have thoroughly examined the thermal properties of natural fibre composites, even though earlier studies have mostly concentrated on the pyhsical behaviour of these materials, notably in terms of ductile and flexural properties^5,38^. This study is among the first to integrate these potent computational tools for thermal optimization, with a particular emphasis on the reinforcing of coir fibres, a sustainable and renewable material. Since the Cuckoo Search Algorithm provides better convergence rates and solution correctness than other conventional optimization techniques, its incorporation in this setting is very novel. This combination of cutting-edge algorithms and material selection advances our knowledge to create natural fibres for high-strength, thermally stable applications.

While previous research on natural fibre-reinforced composites has primarily focused on enhancing mechanical properties such as tensile strength, impact resistance and wear performance, limited attention has been paid to systematically optimising their thermal characteristics, particularly for composites utilizing coir fibre as reinforcement. Moreover, most existing studies have relied on traditional optimization techniques or focused on single-algorithm approaches, often overlooking the complexity involved in multivariable thermal behaviour. This study distinguishes itself by integrating Response Surface Methodology (RSM) with three advanced bio-inspired optimization algorithms such as Particle Swarm Optimization (PSO), Dragonfly Optimization (DFO) and Cuckoo Search Algorithm (CSA) to comprehensively optimize the thermal conductivity of coir fibre-reinforced PVC composites. Notably, the use of CSA in this context is novel and demonstrates superior performance in terms of convergence and accuracy compared to other techniques. By combining robust statistical design with intelligent optimization algorithms, this work offers a more precise and efficient pathway to tailor thermal properties for sustainable, high-performance composite applications. This approach to optimization addresses an important research need and improves the practical use of coir fibres in managing heat in the construction, electronics and automotive industries.

In conclusion, this research aims to further enhance our understanding of the thermal characteristics of PVC reinforced with coir fibres and to optimize these properties by utilizing state-of-the-art computational techniques. Through the integration of RSM, PSO, DFO and CSA, this study offers significant contributions to the field of sustainable, high-performance material design. The results of this investigation should advance the area of composite materials in general and open new avenues for the application of natural fibres in engineering. In this work, PSO is employed to adjust input parameters such as fibre content and chemical treatment levels to optimize the thermal properties of PVC reinforced with coir fibre. Building upon the methodologies of previous studies, this approach aims to enhance the material properties through systematic optimization techniques. By offering a thorough examination of the thermal behaviour of PVC reinforced with coir fibre and concentrating on thermal conductivity, this study seeks to close that gap. This work is unique because it takes a different approach to optimize the thermal characteristics of PVC composites reinforced with coir fibres. Specifically, it combines RSM with sophisticated optimization algorithms such as PSO, DFO and CSA.

## Materials and methods

**Fig. 1:**
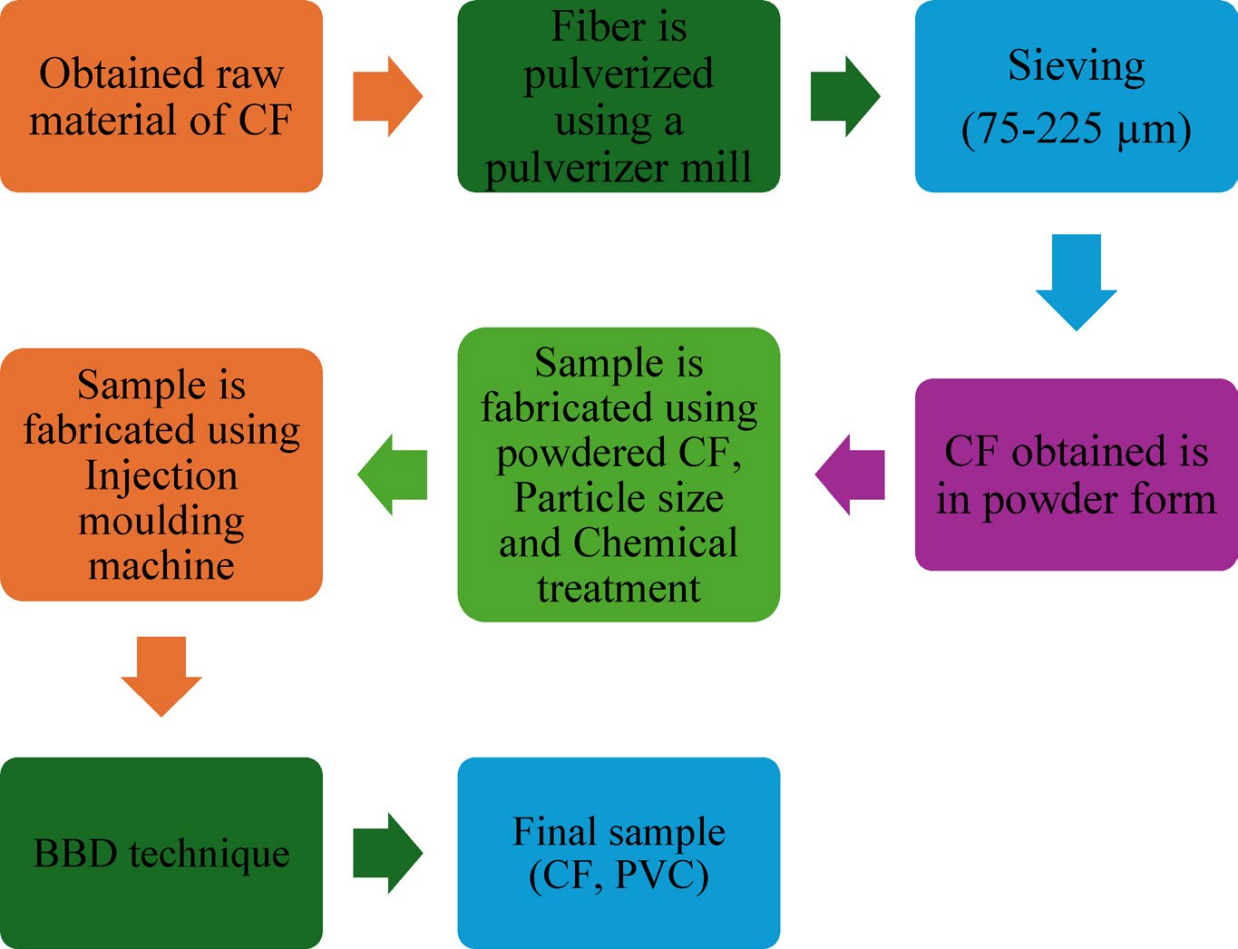
Flowchart of sample fabrication.

In this research, the authors examine the combination of recyclable polymer composites reinforced with coir fibrefibre. The investigation applies three optimization algorithms, namely Particle Swarm Optimization (PSO), Dragonfly Optimization (DFO) and Cuckoo Search Algorithm (CSA), to optimize the reinforcement parameters such as chemical treatment (CT), particle size (PS) (microns) and fibre content (FC) as detailed in Table 1. These algorithms are employed to evaluate their effects on maximizing thermal conductivity. The sample preparation is initiated by splitting the coir fibers into small pieces using scissors. The chopped coir fibers (CF) are then fed into a Pulverizer where the fibers are converted into fine powder. The fine CF powder is isolated by a sieving machine. The coir fiber powder and Polyvinylchloride are fed into heated hopper barrel that improves the heating progression together with the shearing action of the screw as presented in Fig. 1.

**Fig. 2:**
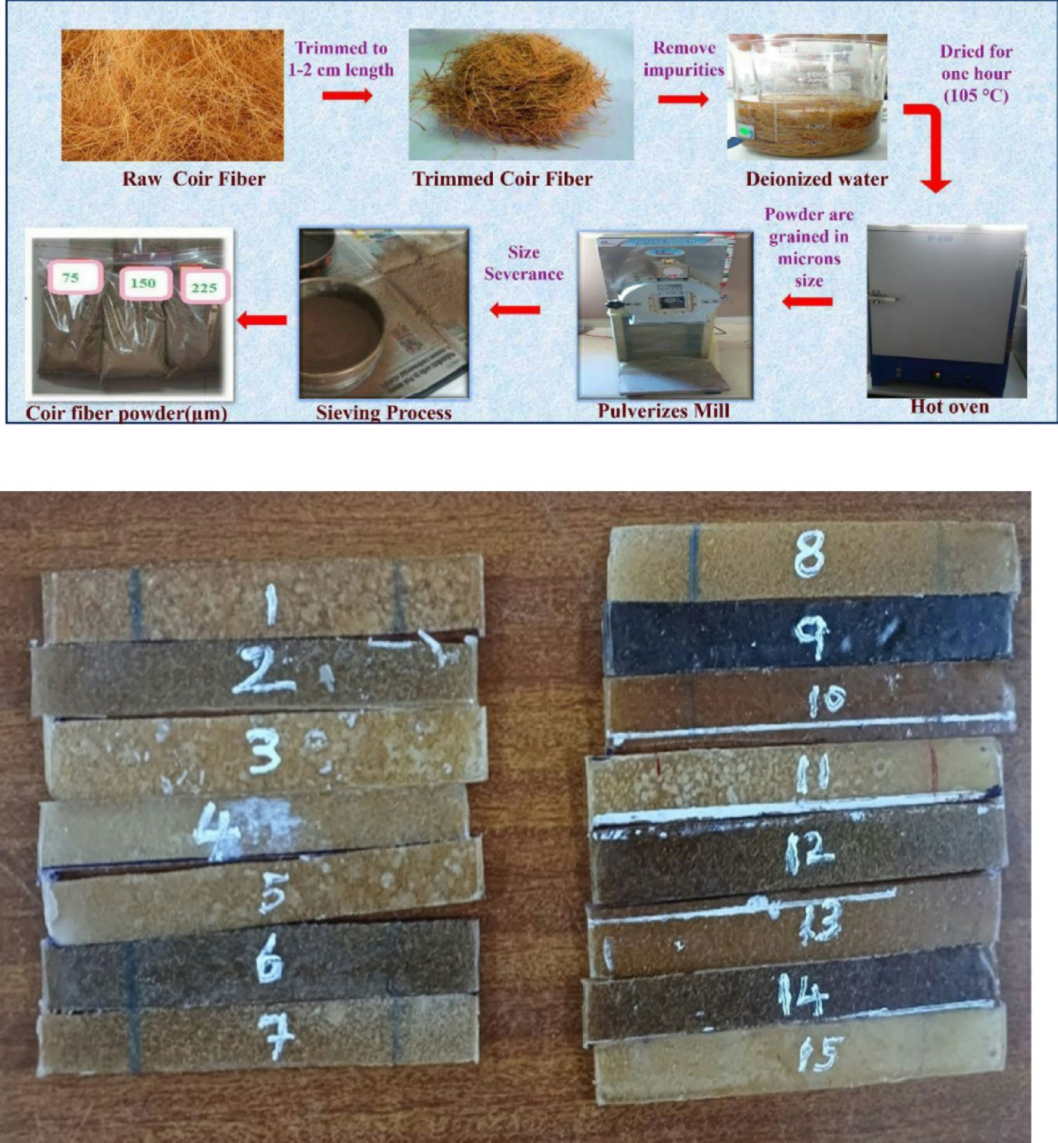
Fabrication of PVC composites.

The experimental setup follows the Box-Behnken design, where coir fibre powder is integrated into PVC to create composite samples. The samples are produced using a hydraulic injection molding machine (Plastics Processing Machine Manufacturer Pvt Ltd, Pune). During the process, coir powder (at a concentration of 2 wt%) and plasticized PVC (98 wt%) are mixed and combined in a cylindrical container by the action of the drive shaft. Once the mixture is adequately sheared, it is injected into the mold. The cooling process begins as soon as the mold is filled, as long as the fastener keeps pressing the polymer-fibre combination. Upon completion of the molding process, composite samples with dimensions of 115 × 95 × 3 mm^3^ are removed from the mold. The procedure is repeated for different fibre weight percentages (4 wt% and 6 wt% coir fibre) as shown in Table 2 and illustrated in Fig. 2.

**Table 1:** Factors, parameters and coded levels of parameters (wt% of CF, PS (µm), CT.

Factor	Parameters	Developed parameter levels		
		−1	0	+ 1
A	FC (wt%)	2	4	6
B	PS (µm)	75	150	225
C	CT	1 (Tri ethoxy (ethyl) silane)	2(Sodium hydroxide)	3 (Potassium Hydroxide)

**Table 2:** Combinations of samples for various parameters using BBD model.

S.No.	FC(wt%)	PS(µm)	CT	K(Wm^− 1^/K)
1	6	150	3	0.769
2	2	150	1	0.735
3	4	150	2	0.782
4	2	75	2	0.768
5	6	225	2	0.788
6	4	225	1	0.720
7	4	225	3	0.788
8	4	150	2	0.789
9	4	75	1	0.777
10	4	75	3	0.780
11	2	150	3	0.779
12	6	150	1	0.780
13	4	150	2	0.786
14	6	75	2	0.769
15	2	225	2	0.788

From the coded level of parameters in the BBD model, fifteen samples are fabricated using the different wt% of CF/PS/CT from Table 2 and these fifteen samples are prepared as per the flowchart represented in Fig. 1.

### Thermal conductivity

**Fig. 3:**
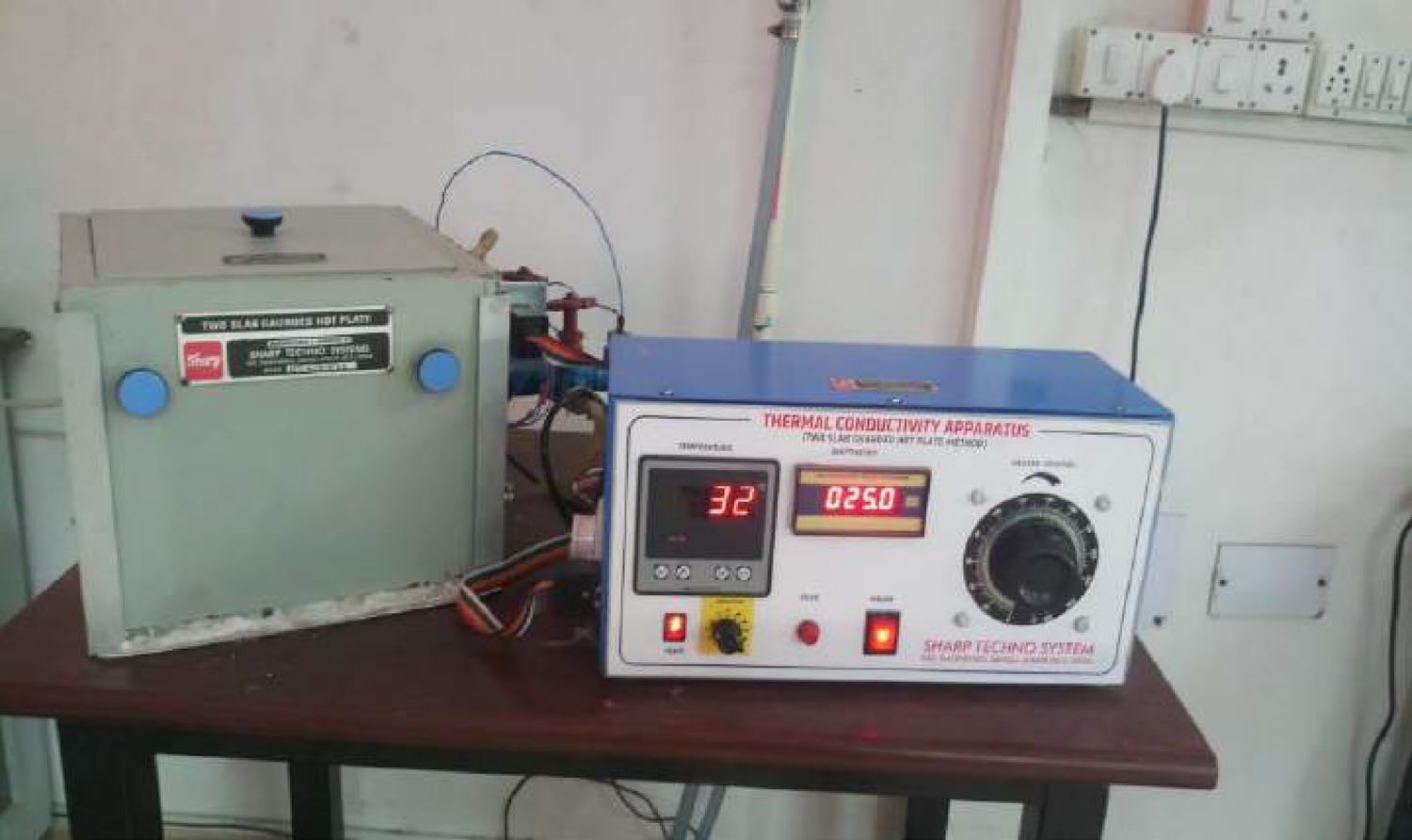
Thermal conductivity apparatus.

**Fig. 4:**
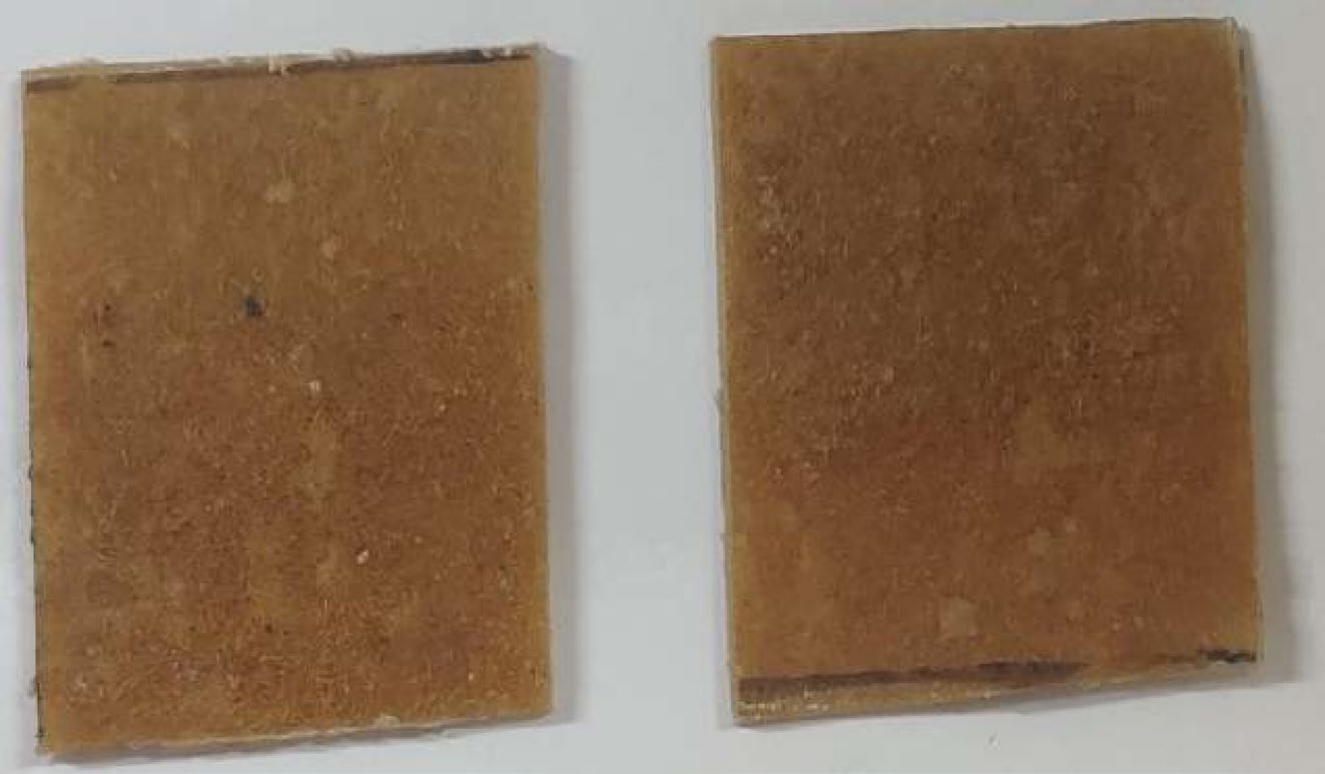
Manufactured specimen: two identical samples.

Two slab-guarded hot plate apparatus (Sharp Techno system, India) is employed to determine the thermal conductivity of composites, as illustrated in Fig. 3. The device is made up of heaters that are both internal and external and are used to produce heat. It also has a temperature indicator, which makes it easier to show a range of temperatures in one place. In addition, the device includes an internal and external heater wattage indicators that allow the amount of heat to be given to be measured. Thermocouples are strategically positioned to ascertain temperature, as delineated below effectively. T1 Thermocouple -The temperature above the primary heating unit, T2 Thermocouple - The temperature exceeded (above) the upper of the specimen, T3 Thermocouple - The temperature under the primary heating unit, T4 Thermocouple - temperature falls below the lower of the samples in Fig. 4. Fourier’s law suggests that all the power applied to the heater travels through the specimen and ultimately finds its way to the isothermal cold plate. The thermal energy that passes through the sample at the elementary level is oriented perpendicular to the lateral guard heater plates’ plane. There is a small gap between the main heater’s measurement area and this guard heater, which encloses it.

### Particle swarm optimization (PSO)

PSO is a population-level metaheuristic procedure stimulated by the collective characteristics of organisms such as bird flocks or fish schools. It works by iteratively refining the location of elements within a search planetary to solve an optimization problem. The individual particle represents a conceivable result and navigates the search space based on its position and velocity, continually adjusting to explore better solutions. The particles share information, enabling collaboration as they seek the optimal outcome^39,40^.

**Fig. 5:**
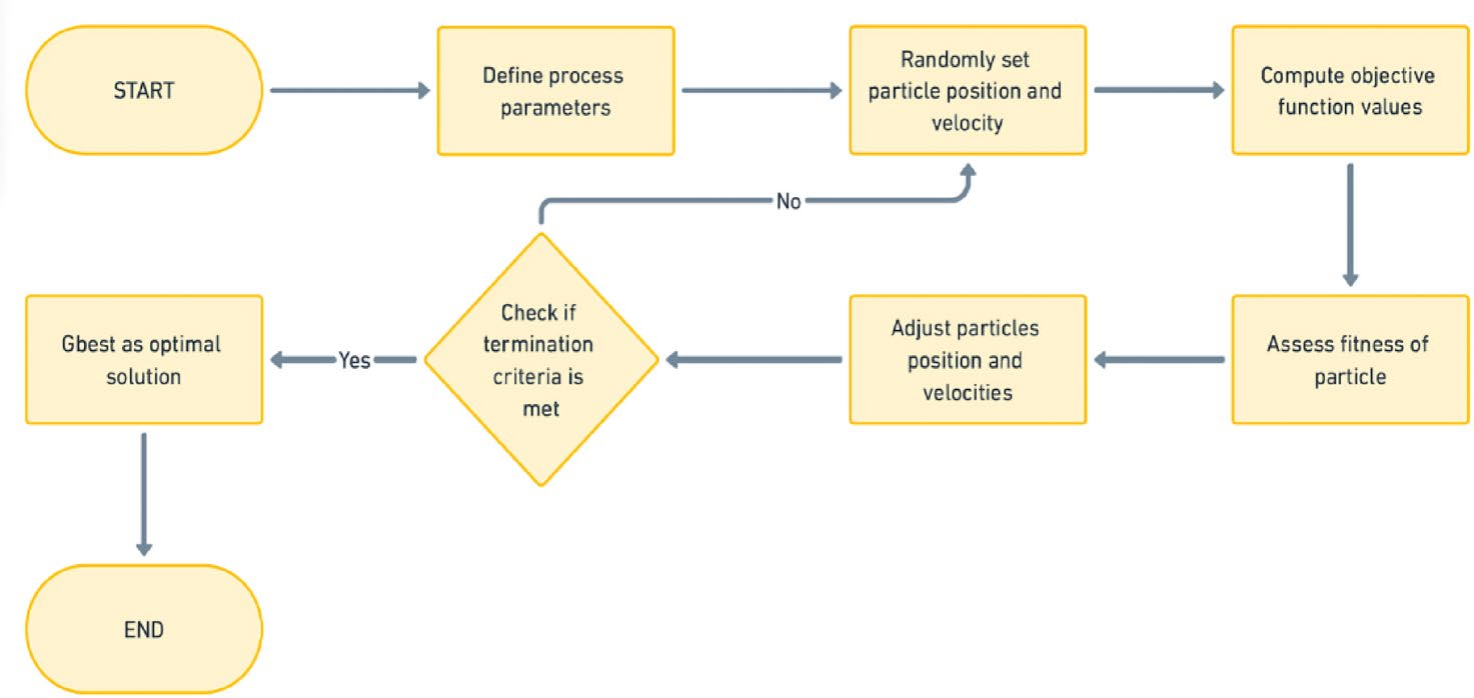
Process of particle swarm optimization.

As illustrated in Fig. 5, the particles update their positions and velocities at each iteration, influenced by both their own past experiences and the performance of the best-performing particles in the swarm. This update process is driven by two key factors: the cognitive element, which steers the element toward its individual best-known position and the societal element, which directs it towards the swarm’s overall best-known position. PSO does not rely on gradient evidence, making it suitable for solving both continuous and discrete optimization problems. Due to its simplicity and computational efficiency, PSO is commonly functional in various fields, including manufacturing, economics and computer science^41,42^.

Nevertheless, PSO has certain limitations, particularly in commerce with multifaceted optimization problems, where it may become trapped in resident optima. To address these challenges, numerous enhancements to the PSO algorithm are proposed, such as incorporating mutation operators, adaptive parameters and hybridization with other optimization techniques^43,44^.

### Dragonfly optimization (DFO)

Dragonfly Optimization (DFO) has emerged as a highly efficient nature-inspired algorithm that surpasses traditional methods like Genetic Algorithms (GA), Ant Colony Optimization (ACO), Simulated Annealing (SA) and Artificial Bee Colonies (ABC) in terms of efficiency, convergence speed, computational complexity and the ability to determine optimal solutions. DFO replicates the crowding behaviour of dragonflies, mimicking the exploratory and exploitative phases of meta-heuristic algorithms. In the exploration phase, also referred to as the stationary swarm, dragonflies form small groups and traverse numerous areas to conduct a search, which is essential for this phase. The stationary swarm dragonflies then converge in a precise direction for more effective exploitation^45^.

DFO has shown remarkable results in optimizing various real-world problems. For example, it has been successfully applied in energy systems like optimizing photovoltaic-fuel cell-hybrid systems, demonstrating better efficiency in system performance. In industrial applications, DFO has been used to optimize combustion processes in coal-fired furnaces, leading to reduced emissions and improved fuel efficiency^46^. Moreover, the algorithm has been adapted to achieve optimal energy consumption in smart grid networks, further highlighting its versatility^47^. To enhance the algorithm’s exploratory capabilities, improvements like the incorporation of Lévy flight strategies have been proposed, allowing better handling of constrained optimization problems and avoiding local optima^48^.

DFO operates through five key phases:

- Phase 1: Random initialization of the dragonfly population, which represents the parameters of the PMC, such as FC, PS and CT.
- Phase 2: Evaluation of the fitness of each dragonfly based on parameters like Young’s modulus, shear modulus, dielectric constant, electrical resistance, impact strength and thermal conductivity.
- Phase 3: This is the cohesion phase, where the dragonflies move collectively, simulating the cohesive behaviour observed in nature.
- Phase 4: Dragonflies update their search positions based on vectors such as food and enemy location. This update is mathematically represented by the Eqs. 1–3^36^:

(a) The change in velocity is represented as: [Eq. 1].

1 $$\:\:{{\Delta\:}\text{V}}_{\text{t}+1}=\text{v}{\text{S}\text{e}\text{p}}_{\text{i}}+\text{a}{\text{A}\text{l}\text{i}\text{g}\text{n}}_{\text{i}}+\text{c}{\text{C}\text{o}\text{h}}_{\text{i}}+\text{f}{\text{F}\text{o}\text{o}\text{d}}_{\text{i}}+\text{e}{\text{E}\text{n}\text{e}\text{m}\text{y}}_{\text{i}}+\text{X}{\Delta\:}\text{P}\text{O}\text{t}$$

Where $\:\text{a}\:$is the weight of alignment, c is cohesion weight, v is separation weight, f is food factor and e is the factor of enemy.

(b) The location of each dragonfly is updated using the equation: [Eq. (2)].

2 $$\:{\text{P}\text{O}}_{\text{t}+1}={\text{P}\text{O}}_{\text{t}}+{{\Delta\:}\text{V}}_{\text{t}+1}$$

- Phase 5: The final phase, referred to as the distraction phase, where the fittest dragonflies (those with optimal fitness values) have a higher probability of achieving better positions. The random walk probability for DFO is described as^37^: [Eq. (3)]

3 $$\:\text{P}=0.3\times\:\left(1-\frac{\text{i}\text{t}\text{e}\text{r}}{\text{i}\text{t}\text{e}\text{r}\:\text{m}\text{a}\text{x}}\:\right)$$

**Fig. 6:**
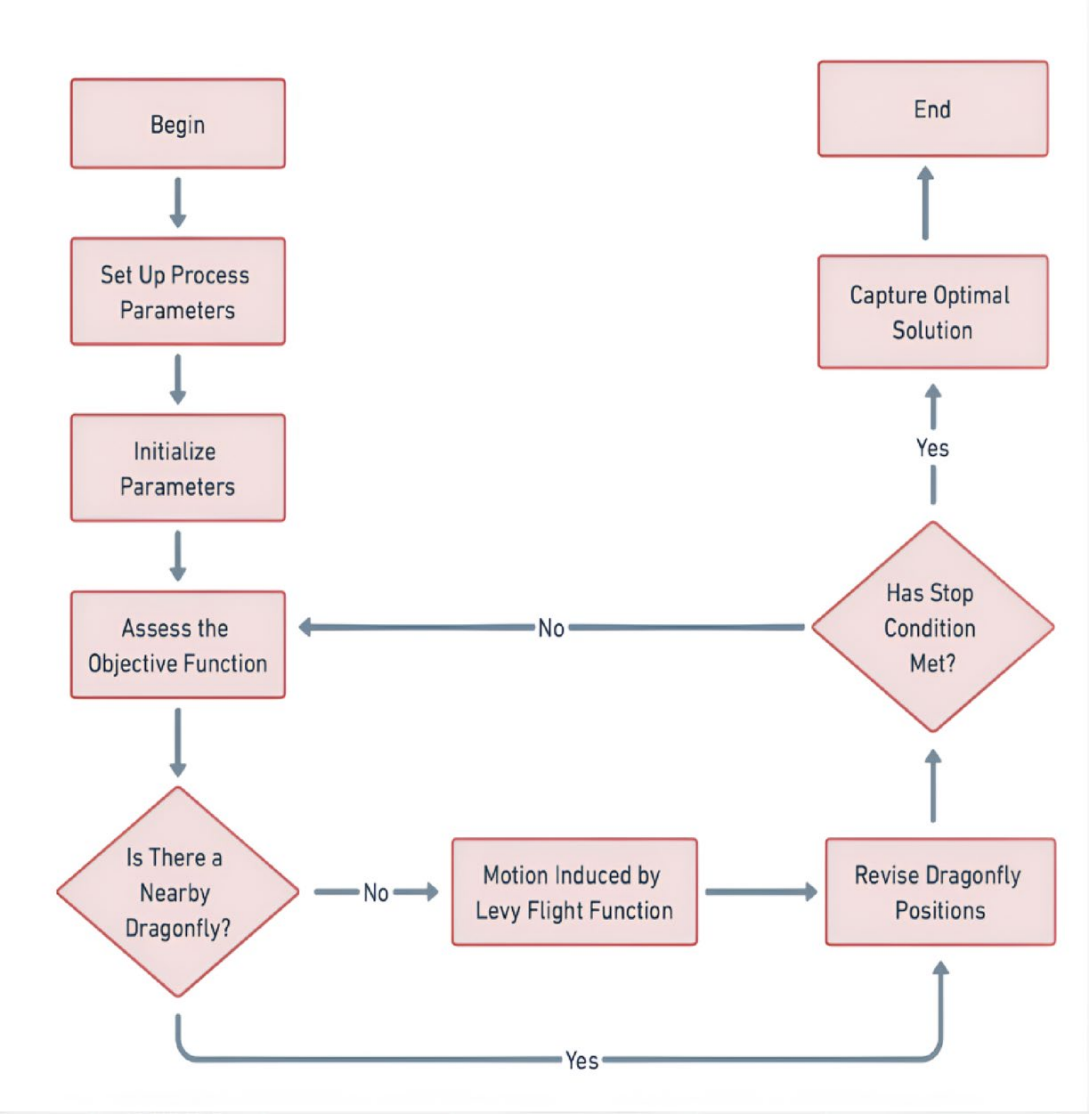
Process of particle swarm optimization.

Figure 6. After the algorithm, the swarm converges into a solitary group, focusing on the best food source and avoiding enemies. The greatest and weakest solutions within the swarm are identified at every iteration, helping the algorithm to refine the solution. The superiority on a global scale is continuously computed and necessary adjustments are made to ensure convergence towards the optimal solution. The DFO model demonstrates a great level of precision in predicting the optimization error and aligns well with experimental data. The fine-tuning of parameters ensures smooth convergence while avoiding issues like premature convergence and local optima.

### Cuckoo search algorithm (CSA)

The CSA is a powerful optimization technique stimulated by the brood parasitism behaviour of certain cuckoo species. These species of birds use another guest species’ nest for laying eggs and if the host bird detects the intruding egg, it either discards the egg or abandons the nest. In optimization terms, this behaviour is analogous to replacing less optimal solutions (or “eggs”) with better ones. CSA mimics this natural phenomenon to efficiently explore the search space, focusing on discovering and retaining the best solutions while discarding the less optimal ones^49,50^.

In CSA, the procedure begins with a people of potential results, or “nests,” which are randomly initialized. The suitability of each nest is assessed according to the unbiased function—in this case, the parameters of the polymer matrix composite, such as FT, PS and CT. A fraction of the nests is then replaced by new, randomly generated solutions if they prove to be more optimal. The process of generating new solutions is modeled after Lévy flight, a random walk with a heavy-tailed probability distribution, which allows CSA to efficiently explore the search space and avoid local optima. This ensures that the algorithm can find global optima more reliably than some other nature-inspired algorithms^51,52^.

The CSA optimization process involves three main stages:

- Phase 1: Initialization, where the algorithm generates a beginning population of nests, in place of the possible solutions to the optimization problem.
- Phase 2: Evaluation and update, where each nest’s fitness is calculated based on composite performance parameters, such as Young’s modulus, shear modulus, dielectric constant, electrical resistance, impact strength and thermal conductivity.
- Phase 3: Lévy flights and nest replacement. A balance between the fraction of the worst-performing nests is maintained by replacing them with new ones generated through Lévy flights. The exploration and exploitation balance is maintained by using a discovery rate parameter $\:{\text{p}}_{\text{a}}$, which determines the fraction of nests to be replaced at each iteration.

It makes use of the following optimization procedures:

1. Initialization: the CSA begins with the initialization of a population of “eggs” representing distinct coefficient sets. Such solutions distribute over the search space that requires wide exploration^53^.
2. Lévy Flight for Exploration: The CSA applies Lévy flights—the random exploratory movements of the algorithm—to various parts of the solution space. This mechanism helps the algorithm to escape local minima, as suggested in the recent applications of CSA in material science^54^.
3. Update population using fitness: the quality of each “egg” or solution is measured in the algorithm as its MSE and lower fitness or higher MSE allows replacing with new ones using the mechanism of natural selection and thereby letting the solutions best fitted evolve at various successive iterations^55^.
4. Convergence and Solution Refining: The algorithm runs 500 iterations to converge the coefficients to the optimal solution. The convergence curve, in terms of the reduction in MSE, has an initial rapid change phase that stabilizes as the solution approaches the global optimum^56^.

The position updates in CSA are influenced by Lévy flights and can be mathematically represented as: [Eq. (4)].

4 $$X_{{t + 1}} = X_{t} + \alpha \times Le^{\prime}vy\left( \lambda \right)$$

where α is the step size, and Lévy(λ) represents the Lévy distribution with step length λ, typically in the range of 1 to 3 for optimization problems.

CSA’s strength lies in its simplicity and its ability to escape local optima, making it highly effective for complex, multi-dimensional problems. Unlike other algorithms that may get trapped in local optima, CSA’s universal search capability allows it to thoroughly explore the solution space. The convergence properties of CSA are particularly beneficial for optimization problems where the search space is rugged or includes many local minima^57,58^.

Despite its effectiveness, CSA also has some limitations. The algorithm’s performance can be sensitive to the discovery rate $\:{\text{p}}_{\text{a}}$​ and fine-tuning this parameter is essential for achieving optimal results. Additionally, CSA can be computationally intensive for very large problems. However, its hybridization with other algorithms or modifications, such as incorporating adaptive Lévy flight parameters, can enhance its efficiency and adaptability to a wider range of optimization challenges^59,60^.

**Fig. 7:**
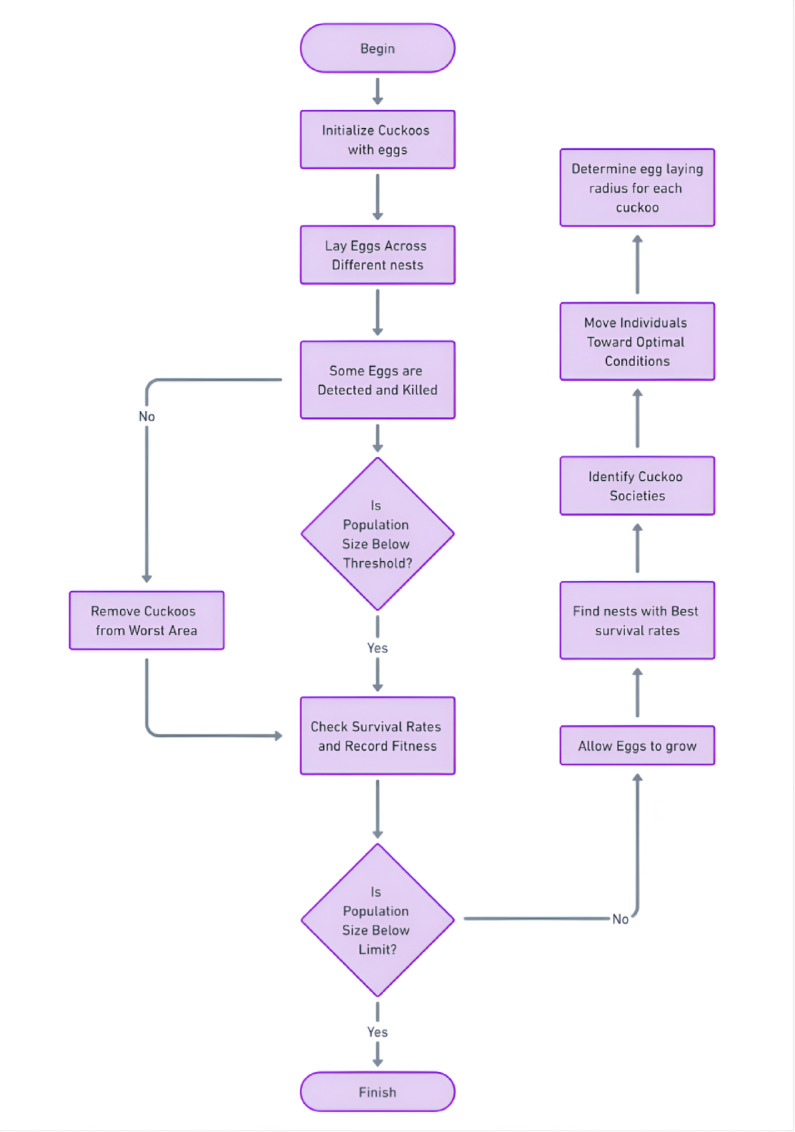
Process of particle swarm optimization.

Overall, CSA provides an efficient and robust approach for optimizing complex problems, including the material properties of fibre-reinforced composites in Fig. 7. In this study, CSA is employed to optimize parameters such as thermal conductivity, dielectric constant and physical characteristics of the coir fibre-reinforced PVC composites, yielding highly accurate predictions and optimal solutions.

## Results and discussions

The thermal conductivity of coir fiber-reinforced PVC composites is significantly influenced by the intrinsic material properties, such as fiber content (FC), particulate size (PS) and chemical treatment (CT). The heterogeneous microstructure of the composite provides complex heat transfer paths. For example, high fiber content can create more interfaces that add to thermal resistance and optimal content maximizes reinforcement and thermal conduction. Particle size and chemical treatment also change the interfacial bonding and fiber dispersion in the PVC matrix. These microstructural changes directly influence heat transfer efficiency and the non-linear interactions among these factors are modeled by the optimization models. Through these interactions, one can understand the following optimization results and implications.

### Optimization of CFRC through PSO

This section tests the parametrized optimization of the coir fibre-reinforced P-PVC composite by the PSO algorithm. The methodology is based on a polynomial model of the CFRC (Coir Fibre-Reinforced Composite). The composite is fabricated through a series of experiments where key parameters - FT (wt%), PS (µm) and CT—are varied and their effects on performance metrics like thermal conductivity (TC) are evaluated. The detailed procedure of the experiment is reported in the published research articles^61,62^. Process parameters are some of the important factors influencing the performance of the coir fibre-reinforced P-PVC composite. Therefore, this study aims to enhance the constraints to ensure that a better-quality product is obtained. The boundaries of the process constraints are tabulated in Table 1.

In the optimization, a polynomial model similar to Response Surface Methodology (RSM) is used, predicting the thermal conductivity based on independent variables X, Y and Z representing fibre content, particulate size and chemical treatments, respectively, along with a set of coefficients. For every iteration, the PSO algorithm optimizes the set of coefficients to minimize prediction errors. The model proves excellent statistical metrics in accuracy, precision, recall and various forms of R-squared (R², adjusted R², and predicted R²), as follows. The error difference in the forecast and investigational values is within the range from 0.06 to 6.54%. Therefore, the model is thoroughly valid. The polynomial regression relation developed through the PSO algorithm for thermal conductivity is defined as: [Eq. (5)]

5 $$\begin{aligned} {\text{TC}} & =0.71374025+0.02712496 \cdot {\text{X}}+0.00382832 \cdot {\text{Y}} \\ & +0.10711503 \cdot {\text{Z}}+0.08073456 \cdot {{\text{X}}^2}+0.02808604 \cdot {{\text{Y}}^2} - 0.06732437 \cdot {{\text{Z}}^2} \\ & - 0.10273473 \cdot {\text{X}} \cdot {\text{Y}} - 0.04496999 \cdot {\text{X}} \cdot {\text{Z}}+0.03422729 \cdot {\text{Y}} \cdot {\text{Z}} \\ \end{aligned}$$

Where X, Y and Z represent FT (wt%), PS (µm) and CT, respectively.

Therefore, the optimization is multi-objective; that is, several performance criteria are trade-balanced for the optimal attainable conditions. This present study uses thermal conductivity as the principal performance measure for the improvement of the desired variable under the PSO algorithm applied to Python. The optimum found by the multi-objective approach of the PSO method is better than those based on a desirability-based optimization method. Table 3 lists the optimal parameters for experiment 13 determined by this implementation of PSO using Python.

**Table 3:** Optimal parameters for PSO.

Run no	Coir powder (wt%)	Particle size (µm)	Surface treatment	Thermal conductivity		Error%
				(W/(m·K))		
				Expected	Predicted	
1	6	150	3	0.769	0.791	2.87
2	2	150	1	0.735	0.723	1.68
3	4	150	2	0.782	0.765	2.20
4	2	75	2	0.78	0.750	3.79
5	6	225	2	0.788	0.782	0.74
6	4	225	1	0.779	0.728	6.54
7	4	225	3	0.78	0.780	0.06
8	4	150	2	0.789	0.765	3.07
9	4	75	1	0.72	0.747	3.82
10	4	75	3	0.788	0.765	2.95
11	2	150	3	0.788	0.780	1.07
12	6	150	1	0.761	0.779	2.39
13	4	150	2	0.786	0.765	2.70
14	6	75	2	0.777	0.836	7.57
15	2	225	2	0.768	0.799	4.10

This analysis shows that PSO can be regarded as an efficient optimization tool when used for the optimization of such complex, multi-variable systems like coir fibre-reinforced P-PVC composite mainly because performance is improved with more accuracy towards predictive modeling. Optimization of process parameters directly reflects upon the thermal conduction optimization of the amalgamated material that relates to the objectives of the current study.

### Optimization of CFRC through DFO

The DFO algorithm solves the optimization problem described in Table 4. In this methodology, a polynomial regression model predicts thermal conductivity based on three input variables: X, Y and Z, which correspond to defined parameters. The model expanded these variables with quadratic terms as well as interaction terms having distinct coefficients for each coefficient in quantifying its influence on the output thermal conductivity. Here the main purpose is to optimize these coefficients for accurate predictions using regularization to prevent overfitting in models. It is demonstrated by the composite test function that the DFO algorithm is competitive and performs better than PSO algorithms on occasion. P values show that superiority is less important than test functions. This is because algorithms utilized in this work have trouble with composite test functions. The suggested DFO algorithm’s performance is examined using four additional measures in the following paragraphs. This experiment tests convergs and predicts DFO algorithm behaviour in real problems. Dragonfly position (search history), parameter value (trajectory), average fitness and optimal food source fitness are quantitative metrics.

In the Dragonfly Optimization algorithm, the location of every particle is iteratively efficient using inertia, cognitive, social components, etc. This method provides an effective balance between individual exploration and alignment with the global optimal solution, thereby further improving the accuracy and reliability of the predictions. The balanced exploration and exploitation enable the optimization of polynomial model parameters based on the DFO algorithm and improve predictive performance for thermal conductivity.

In the subsequent sections, four supplementary performance measures of the proposed Dragonfly Optimization (DFO) algorithm are used to further assess its effectiveness. It analyzes the convergence rate and predictive reliability of the DFO algorithm in handling challenging optimization problems. In this context, four quantitative measures are monitored viz., dragonfly positions or code for the search history, values of parameters as trajectory, mean fitness of the population and fitness value of the optimal food source. The model iterates 2000 times to maximize predictive precision. In the early iterations, drastic changes are noticed, which eventually become negligible with iterations. According to Berg et al.^63^, this behaviour indicates that the algorithm tends to converge to an optimal region in the search space, thus providing efficient search behaviour and convergence. The polynomial regression model for thermal conductivity, as obtained using the PSO algorithm, is given as: [Eq. (6)].

**Table 4:** Optimal parameters for DFO.

Run no	Coir powder (wt%)	Particle size (µm)	Surface treatment	Thermal conductivity		Error%
				(W/(m·K))		
				Expected	Predicted	
1	6	150	3	0.769	0.786	2.24
2	2	150	1	0.735	0.711	3.29
3	4	150	2	0.782	0.769	1.65
4	2	75	2	0.78	0.731	6.26
5	6	225	2	0.788	0.790	0.25
6	4	225	1	0.779	0.773	0.81
7	4	225	3	0.78	0.789	1.12
8	4	150	2	0.789	0.769	2.52
9	4	75	1	0.72	0.707	1.82
10	4	75	3	0.788	0.789	0.17
11	2	150	3	0.788	0.787	0.15
12	6	150	1	0.761	0.764	0.37
13	4	150	2	0.786	0.769	2.15
14	6	75	2	0.777	0.769	1.09
15	2	225	2	0.768	0.775	0.90

6 $$\begin{aligned} TC & =0.67207208+0.07508651 \cdot X+0.07796376 \cdot Y \\ & +0.12707956 \cdot Z - 0.01093291 \cdot {X^2} - 0.00098001 \cdot {Y^2} - 0.01787645 \cdot {Z^2} \\ & - 0.02233237 \cdot X \cdot Y - 0.05355997 \cdot X \cdot Z - 0.06641321 \cdot Y \cdot Z~ \\ \end{aligned}$$

Where, X, Y and Z, denote FT (wt%), PS (µm) and CT, respectively.

The DFO algorithm activities the examination space as exemplified by the change of dragonflies’ locations for optimization. Monitoring parameter values across iterations provides insight into the development of potential solutions. Ideally, the characteristics of exploration and exploitation should vary, with abrupt changes early on, followed by more gradual adjustments. This balance is critical, as it enables the swarm to collectively improve in fitness over time. Ultimately, the fitness of the food source—representing the global optimum—continues to improve as the optimization progresses. Throughout testing, the average fitness of the dragonfly population improves consistently across all test functions, which indicates an improvement in the initial random population’s fitness.

The same tendencies are found in the convergence curves and show that the estimate of the comprehensive optimum strengthens with each iteration. Most striking is the accelerations in these convergence curves, showing better localization and exploitation of the search space as the algorithm approaches the optimum.

In the final phases of the process, this acceleration trend leads to convergence toward the global optimum solution at a rapid pace. This analysis illustrates the way the proposed DFO algorithm balances between exploration and exploitation. With this, adaptive behaviour enhances the convergence property of a dragonfly swarm towards global optima at further iterations. The pseudocodes given in Table 5 of the DFO algorithm define its parameter conditions explaining the robustness and adaptability of this optimization technique.

### Optimization of CFRC through CSA

Optimization of Thermal Conductivity for Coir Fibre-Reinforced P-PVC Composite (CFRC) using the Cuckoo Search Algorithm (CSA) as presented in Table 5, the Cuckoo Search Algorithm is utilized to optimize the coefficients of a polynomial model estimating thermal conductivity based on input features X, Y and Z, which represent fibre content, particulate size and chemical treatment. This optimization technique mimics the parasitic egg-laying behaviour of certain species of cuckoos. Adding quadratic and interaction terms based on other related optimization studies^64,65^, this polynomial model captures each variable’s effect on thermal conductivity. To put it briefly, CSA aims at the optimization of such models towards minimizing MSE-the deviation of predicted values from real values.

The polynomial regression model of thermal conductivity, obtained from the PSO algorithm, is given in: [Eq. (7)].

7 $$\begin{aligned} TC & =0.42119008+0.44336437 \cdot X - 0.37151552 \cdot Y \\ & +0.96023282 \cdot Z - 0.27536445 \cdot {X^2}+0.2417812 \cdot {Y^2} - 0.37423457 \cdot {Z^2} \\ & +0.30903895 \cdot X \cdot Y - 0.64947781 \cdot X \cdot Z+0.30740514 \cdot Y \cdot Z \\ \end{aligned}$$

**Table 5:** Optimal parameters for CSA.

Run no	Coir powder (wt%)	Particle size (µm)	Surface treatment	Thermal conductivity		Error%
				(W/(m·K))		
				Expected	Predicted	
1	6	150	3	0.769	0.769	0.01
2	2	150	1	0.735	0.740	0.74
3	4	150	2	0.782	0.777	0.63
4	2	75	2	0.78	0.776	0.55
5	6	225	2	0.788	0.788	0.01
6	4	225	1	0.779	0.761	2.27
7	4	225	3	0.78	0.801	2.64
8	4	150	2	0.789	0.777	1.51
9	4	75	1	0.72	0.760	5.50
10	4	75	3	0.788	0.778	1.32
11	2	150	3	0.788	0.791	0.44
12	6	150	1	0.761	0.763	0.22
13	4	150	2	0.786	0.777	1.14
14	6	75	2	0.777	0.765	1.55
15	2	225	2	0.768	0.777	1.22

The model coefficients from the optimized CSA yield very accurate prediction of thermal conductivity with minimum MSE through iterative refinement. For each set of optimized coefficients obtained from CSA, the predictions for the thermal conductivity of the experimental data points are calculated and the percentage error for each prediction is obtained The accuracy of the model’s predictions is indicated by the percentage error. Lower percentages indicate a higher reliability. Furthermore, the convergence pattern, as shown in the convergence graph, reflects that the algorithm can reach a global optimum since MSE values drop sharply with time.

### Comparative analysis of the PSO‑DFO‑CSA algorithm

**Fig. 8:**
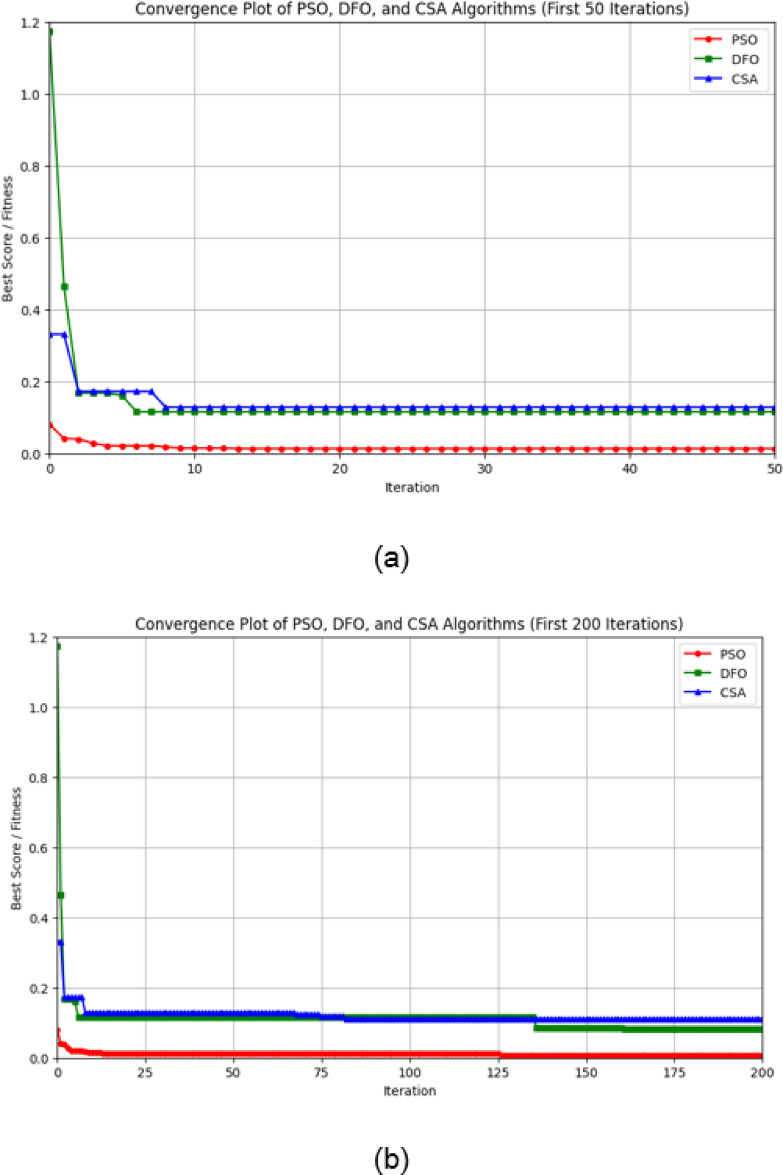
(**a**) Convergence plot of PSO, DFO and CSA algorithm 50 iterations. (b) Convergence plot of PSO, DFO and CSA algorithm 200 iterations.

The convergence behaviour of PSO, DFO and CSA is very interesting since it gives insight into optimization characteristics and effectiveness for the various iteration counts. PSO converges at the very beginning of the initial stage, which is within the first 50 iterations as it can be seen in Fig. 8a. Since it shows rapid convergence to a low error value, then its capacity for high-speed optimization and stability in reaching the near-optimal solution is great. DFO follows the same path in having an initial rapid fall in error but does level off at a little bit higher error value as that of PSO. On the other hand, the gradual decrease in error during this stage is seen for CSA while demonstrating a more conservative type of convergence in comparison with the other two algorithms.

In Fig. 8b, when the convergence is extended to 200 iterations, PSO keeps low error consistently by strengthening its efficiency in achieving stable solutions with minimal fluctuations. On an interesting note, the DFO and CSA converged closely as iterations moved past 100 and both attained similar error levels around the 125th iteration. This implies that even though CSA begins converging slower than DFO, CSA regains the accuracy of DFO for additional iterations and eventually gets stabilized to a level close to that of DFO. All these results point towards recommending PSO for applications requiring fast convergence. DFO and CSA are very good alternatives when further iterations are allowed to achieve comparable accuracies. The maximum thermal conductivity is 0.801 $\:\left(\frac{W}{mK}\right)$ by the Cuckoo Search Algorithm (CSA).

**Fig. 9:**
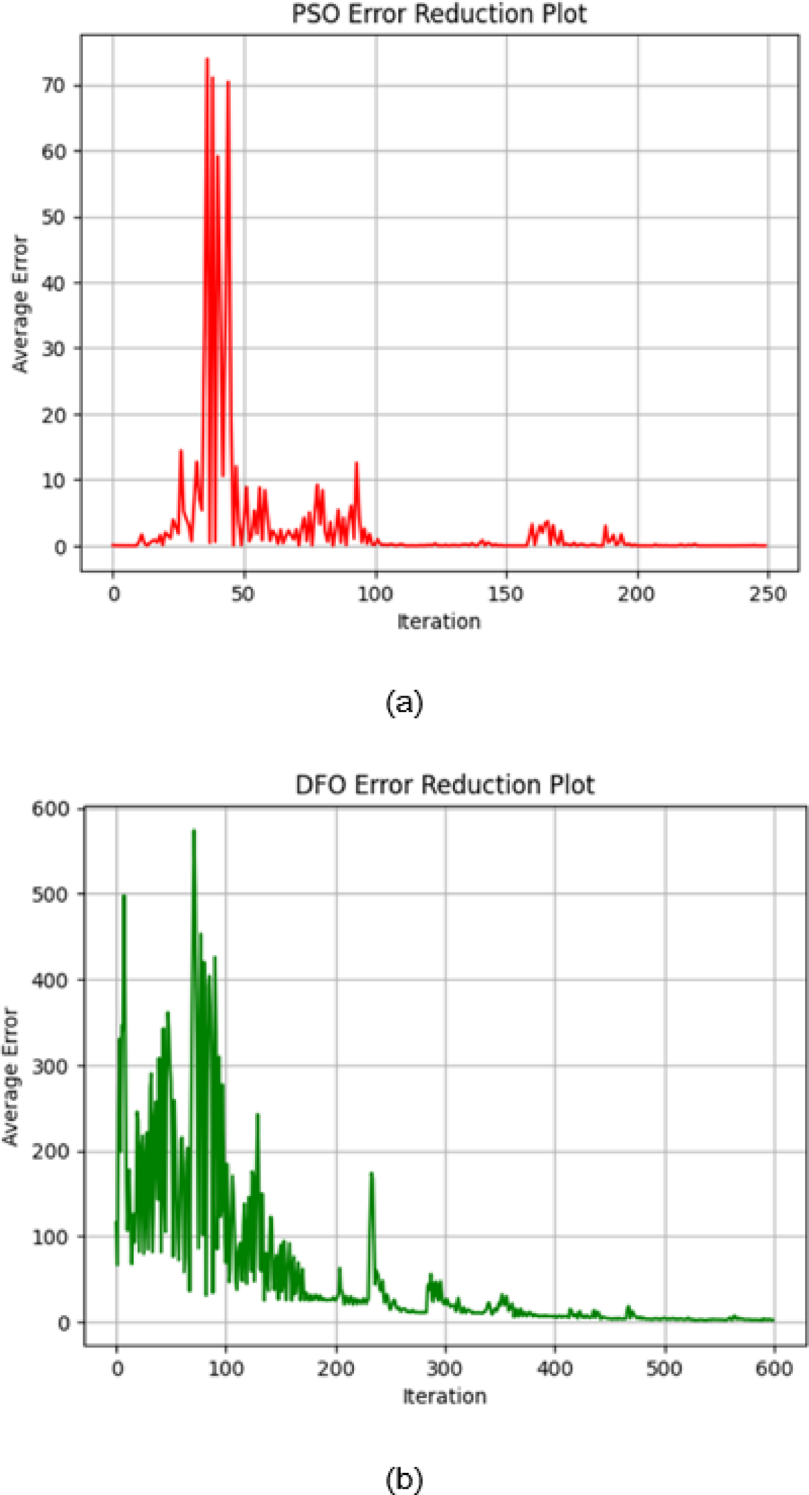

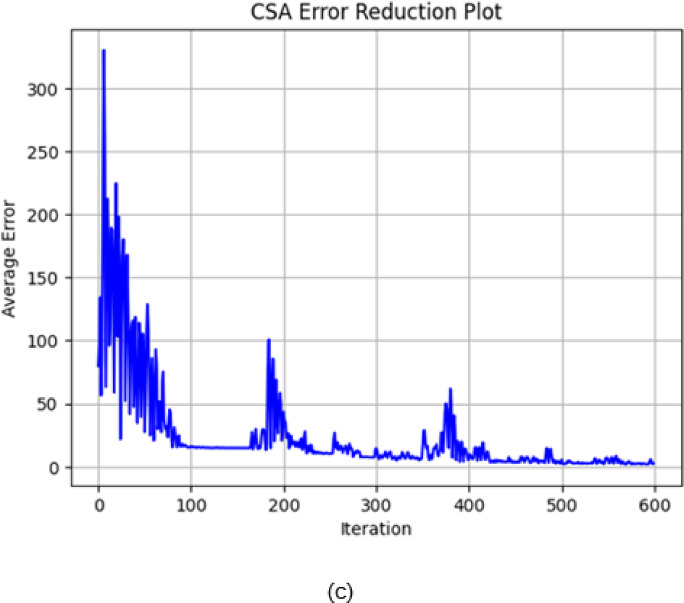
(**a**) Error reduction plot of PSO. (**b**) Error reduction plot of DFO. (**c**) Error reduction plot of CSA.

Figure 9a shows the error reduction graph of PSO illustrating a very sharp initial decrease in error, but it has sharp spikes during the early iterations. Oscillations decrease and become stable after 150 iterations when the error remains near zero and fluctuations are negligible afterwards. It means that the algorithm is struggling at first but recovers well to converge at a low level of error with stable behaviour throughout the remaining iterations.

PSO updates the velocity and position of each particle from its own best-known position and the swarm’s best-known position. This provides rapid convergence in the early iterations, where particles move quickly towards promising areas in the search space. But this rapid movement also tends to lead to premature exploitation, i.e., after the particles move close to each other, they are trapped in local minima. This can be seen from the early steep drop in errors and occasional spikey peaks in error (e.g., in Fig. 9a), which indicates that although PSO performs well for rapid optimization, its rapid convergence comes at the cost of oscillations in fine-tuning the optimal solution.

On the other hand, in Fig. 9b, DFO starts with significantly higher error values and shows marked oscillations during the initial phase, especially in the first 100–150 iterations. In the latter, the error-decline process is gradual, though convergence is slower. Oscillations continue throughout the iterative process, indicating that the latter algorithm may require more iterations to have significant values of reduced error and less stability in the long run.

The Dragonfly Optimization algorithm balances exploration and exploitation by mimicking the natural dragonfly behaviours—through cohesion, separation and alignment. DFO initializes with a longer time exploring the search space and hence the error values are large and oscillatory (as observed in Fig. 9(b)). This extended exploration period prevents DFO from converging towards local optima. Since the algorithm is constantly refining the direction of search using a increasing number of iterations, a consistent reduction in error is achieved. This is particularly beneficial while dealing with complex multimodal optimization landscapes such that a consistent convergence is achieved that captures the complexity of the composite thermal behaviour.

CSA’s error reduction, as shown in Fig. 9c, starts very high but can be observed to come down with iteration. Periodic spikes of the same order as DFO are seen. These decrease with advancing iteration and the error stabilizes around 400–600 iterations. It can be noted that though oscillating all through the earlier and middle periods, this reduction is much more orderly after a lengthier time of iteration and finally stabilizes to a low value compared to the second algorithm with fewer oscillations as well.

CSA is motivated by the Lévy flight concept, where large random steps enable the algorithm to explore distant areas of the search space. CSA also exhibits a slightly more gradual reduction in initial errors (as in Fig. 9(c)) and this process prepares it well to escape from local minima and achieve a global optimum at a smaller mean squared error (MSE) subsequently. The periodic spikes in the trajectory of intermediate iterations are a direct result of its stochastic search nature. As iterations go further, the error converges to a lower value, confirming CSA’s high ability to improve the solution in a rugged high-dimensional search space.

Although all three algorithms have different tendencies for initial error reduction and fluctuation patterns, they can minimize error over time with a difference in rates and levels of stability. PSO shows a faster reduction in error with greater stability, while DFO and CSA need more steps to reach comparable reductions in the error.

The scatter plots are a great way to illustrate the relationship between experimental (actual) and predicted thermal conductivity values for three optimization algorithms. The ideal performance (y = x line) is indicated by the red dashed line, where perfect predictions would align.

**Fig. 10:**
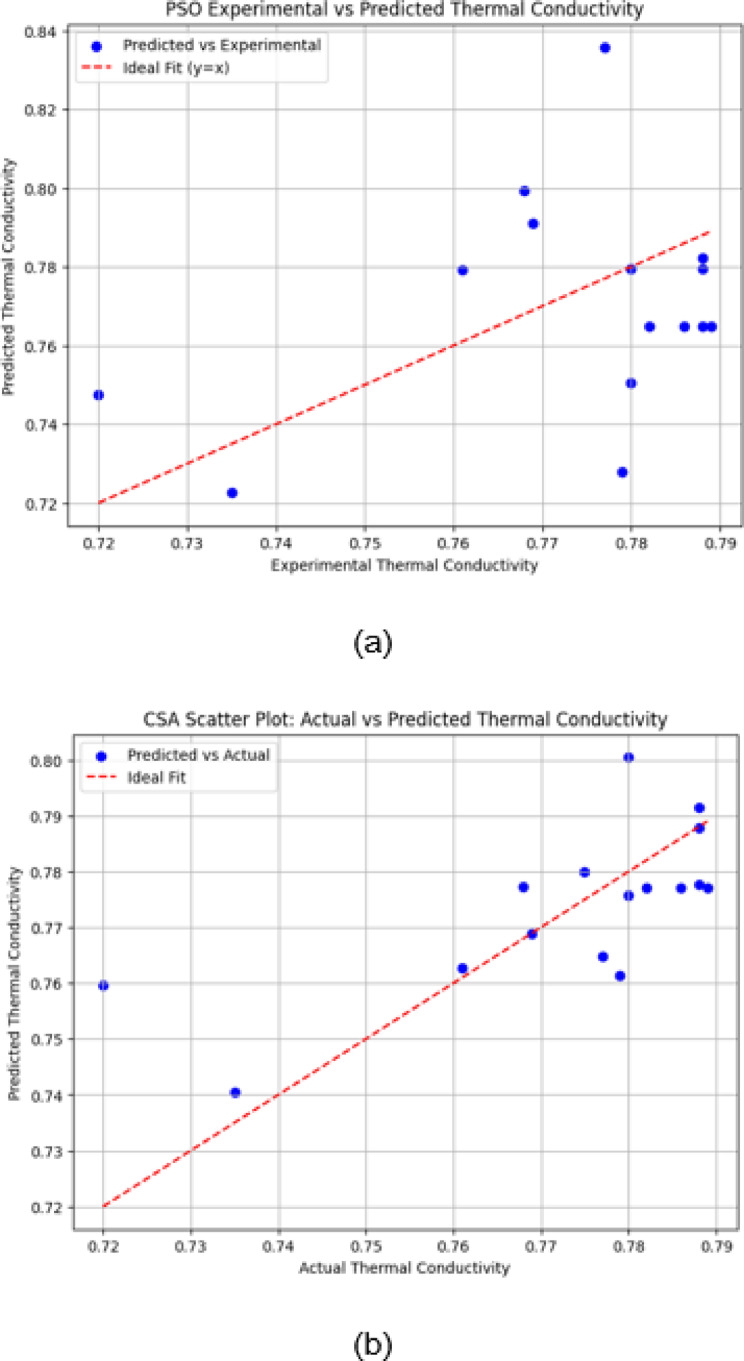

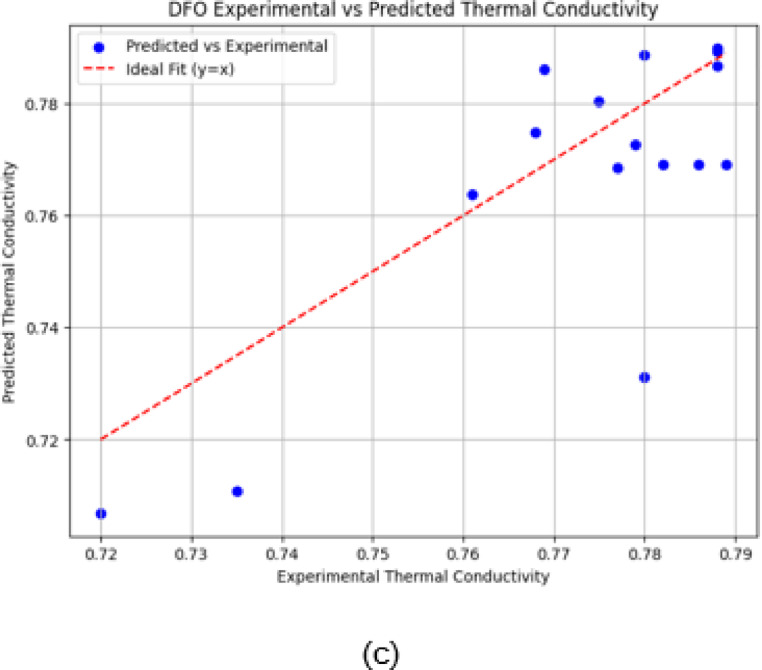
(**a**) Predicted vs. experimental values using PSO. (**b**) Predicted vs. experimental values using CSA. (**c**) Predicted vs. Experimental values using DFO.

The scatter plot of the PSO-based model (shown in Fig. 10a) demonstrates a higher spread of predicted values, particularly for experimental conductivities above 0.76. Several data points deviate significantly from the ideal line, suggesting that PSO exhibits less accuracy and higher variance in its predictions compared to CSA.

The scatter plot of CSA-based model (shown in Fig. 10b) illustrates relatively close predictions to the experimental values, with most points clustered near the ideal line. However, slight deviations are observed, particularly at lower and higher conductivity values.

The DFO-based model performs better than PSO, with predictions more tightly clustered around the ideal line in Fig. 10c. However, minor deviations still occur at lower and intermediate conductivity values. Compared to CSA, DFO shows slightly improved prediction alignment, with fewer outliers and better generalization across the experimental range.

The scattering of data points in the PSO scatter plot (Fig. 10(a)) shows greater variability in predictions, particularly at higher thermal conductivity, suggesting that PSO’s rapid convergence might overlook some nuances in the data. In contrast, the tighter clustering near the ideal line in the CSA scatter plot (Fig. 10(b)) confirms its superior accuracy. The intermediate performance depicted in the DFO scatter plot (Fig. 10(c)) confirms its position as a well-balanced optimizer.

The observed behaviour in the optimization outcomes is justified by theoretical models and the current literature in the field. PSO’s convergence rate has been extensively documented in swarm intelligence literature, where the balance between cognitive and social terms results in high-speed movement but can lead to premature convergence. Likewise, literature for DFO shows that it is effective in searching multimodal functions by preserving diversity in the search process. CSA’s capacity to escape local optima through Lévy flights is in line with current studies that prove it to be superior in the case of complex optimization problems. Coupling these with our experimental results further justifies the technical basis of our optimization approach and confirms the differences in behaviour observed.

The comparative analysis of PSO, DFO and CSA highlights the distinct advantages and trade-offs among these optimization algorithms for enhancing the thermal conductivity of coir fibre-reinforced PVC composites. PSO exhibits rapid convergence and stability, making it highly effective for applications where quick optimization is essential. DFO, while slower in convergence, demonstrates strong exploratory capabilities, allowing it to navigate complex search spaces effectively. CSA, on the other hand, provides the best overall accuracy in predicting thermal conductivity, despite requiring more iterations to reach optimal solutions. The choice of algorithm ultimately depends on the specific requirements of the optimization task—whether prioritizing speed, accuracy or robustness. This study reinforces the significance of metaheuristic optimization in composite material design and suggests that hybrid approaches integrating these algorithms could further enhance optimization efficiency for future applications.

**Fig. 11:**
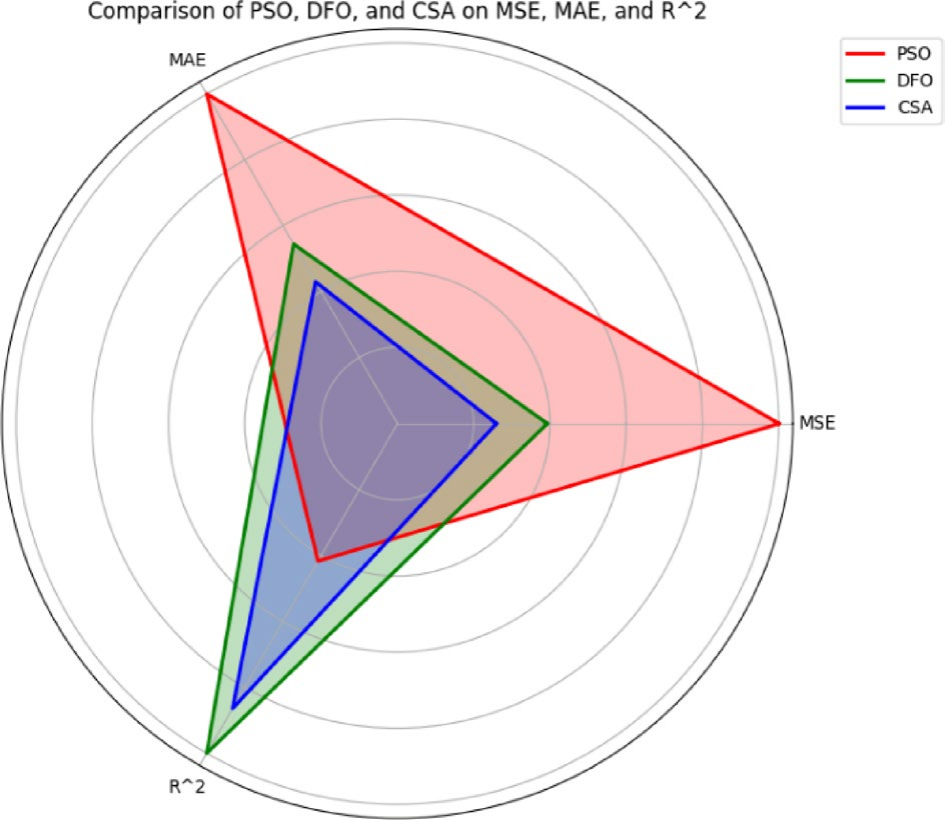
Radar chart for comparison of PSO, DFO and CSA.

In the Radar chart shown in Fig. 11, a comparison is made based on performance from three evaluation metrics-mean squared error, mean absolute error and coefficient of determination. $\:{R}^{2}$ between three optimization algorithms-Particle Swarm Optimization, Dragonfly Optimization and Cuckoo Search Algorithm. In this graph, the error and correlation values are plotted in a way that forms a triangular shape. The larger the error indicates the longer the sides of the triangle. Three triangles of different colours illustrate the performance of the three optimization algorithms. The smallest area triangle is the best model. Therefore, it can be seen that CSA is the best-performing model, DFO performs reasonably well and PSO performs the worst.

## Conclusion

This research systematically investigates the optimization of thermal conductivity in coir fibre-reinforced polyvinyl chloride (PVC) composites using advanced metaheuristic algorithms Cuckoo Search Algorithm (CSA), Dragonfly Optimization (DFO) and Particle Swarm Optimization (PSO) in conjunction with Response Surface Methodology (RSM). The comparative analysis reveals that CSA achieves superior predictive accuracy, yielding the highest optimized thermal conductivity value of 0.801 W/mK by the process parameters such as potassium hydroxide treatment, coir content of 2 wt% and powder diameter of 75 (µm). Additionally, CSA exhibits the lowest Mean Squared Error (MSE) and Mean Absolute Error (MAE), with error deviations between experimental and predicted results ranging from 0.01 to 5.5%. These metrics indicate CSA’s strong convergence behaviour and high approximation fidelity.

DFO demonstrates a slightly broader error margin (0.15–6.26%) but attains a higher coefficient of determination (R²), suggesting that it effectively captures data variance and is well-suited for applications prioritizing statistical model robustness. Conversely, PSO presents higher MSE and MAE values, along with a comparatively lower R², indicating reduced correlation with experimental data and greater prediction uncertainty. Despite its efficient convergence properties, PSO is less reliable in terms of modelling accuracy for thermal property prediction.

From a practical standpoint, the findings underscore the applicability of CSA and DFO as robust optimization tools in the domain of thermally engineered bio-composites. CSA is particularly advantageous in scenarios requiring high-precision parameter tuning, while DFO is suitable where consistency and model generalization are paramount. The optimized coir-PVC composites, developed through this methodology, exhibit enhanced thermal performance, making them viable for thermal insulation and energy-efficient applications in the construction, electronics and automotive sectors. Moreover, the integration of natural coir fibre supports sustainable material development by reducing reliance on synthetic reinforcements. This study provides a comprehensive computational framework for multi-objective optimization of thermophysical properties in polymer composites, advancing the state of research in eco-efficient composite design.

There is a wide scope of research in this area and some of the scopes for future works includes conducting research on vibration and acoustic analysis of composites reinforced with coir fibers and developing the product for use in electrical devices such as switches and electronic chips for electric boards.

## Electronic supplementary material

Below is the link to the electronic supplementary material.

Supplementary Material 1

## Data Availability

The datasets used and analyzed during the current study are available from the corresponding author on reasonable request.
