# Supplementary material for: Optimization of thermal conductivity in coir fibre-reinforced PVC composites using advanced computational techniques
Source: Sci Rep. 2025 May 14;15:16675. doi: 10.1038/s41598-025-01471-8 (PMC12078578; doi:10.1038/s41598-025-01471-8)
Supplement: Supplementary file 1 — Supplementary Material 1 [file 41598_2025_1471_MOESM1_ESM.docx]

**Supplementary Material**

**A1.** Python Codes for all the 3 provided below:

PSO

import numpy as np

import matplotlib.pyplot as plt

# Experimental data is feeded here inside the model____

experimental_data = np.array([

[6, 150, 3, 0.769],

[2, 150, 1, 0.735],

[4, 150, 2, 0.782],

[2, 75, 2, 0.780],

[6, 225, 2, 0.788],

[4, 225, 1, 0.779],

[4, 225, 3, 0.780],

[4, 150, 2, 0.789],

[4, 75, 1, 0.720],

[4, 75, 3, 0.788],

[2, 150, 3, 0.788],

[6, 150, 1, 0.761],

[4, 150, 2, 0.786],

[6, 75, 2, 0.777],

[2, 225, 2, 0.768],

])

# Polynomial model for thermal conductivity is defined here____

def thermal_conductivity(X, Y, Z, coeffs):

return (coeffs[0] + coeffs[1] * X + coeffs[2] * Y + coeffs[3] * Z +

coeffs[4] * X**2 + coeffs[5] * Y**2 + coeffs[6] * Z**2 +

coeffs[7] * X * Y + coeffs[8] * X * Z + coeffs[9] * Y * Z)

# PSO parameters are set here____

num_particles = 50

num_dimensions = 10

num_iterations = 500

w_max = 0.9

w_min = 0.4

c1 = 2

c2 = 2

# Particles are intialised here____

np.random.seed(42)

particles = np.random.uniform(-0.5, 0.5, (num_particles, num_dimensions))

velocities = np.random.uniform(-0.05, 0.05, (num_particles, num_dimensions))

# Personal best positions and global best position are initialised here____

p_best_positions = particles.copy()

p_best_scores = np.full(num_particles, np.inf)

g_best_position = np.zeros(num_dimensions)

g_best_score = np.inf

# Normalization function is taken out here____

def normalize(value, min_val, max_val):

return (value - min_val) / (max_val - min_val)

# Experimental data is normalised____

X_min, X_max = experimental_data[:, 0].min(), experimental_data[:, 0].max()

Y_min, Y_max = experimental_data[:, 1].min(), experimental_data[:, 1].max()

Z_min, Z_max = 1, 3

normalized_data = np.copy(experimental_data)

normalized_data[:, 0] = normalize(experimental_data[:, 0], X_min, X_max)

normalized_data[:, 1] = normalize(experimental_data[:, 1], Y_min, Y_max)

normalized_data[:, 2] = normalize(experimental_data[:, 2], Z_min, Z_max)

# Optimization loop is configured____

convergence = []

for iteration in range(num_iterations):

w = w_max - ((w_max - w_min) * (iteration / num_iterations))

for i in range(num_particles):

coeffs = particles[i]

errors = []

for data in normalized_data:

X, Y, Z, exp_val = data

pred_val = thermal_conductivity(X, Y, Z, coeffs)

errors.append((exp_val - pred_val)**2)

score = np.mean(errors)

if score < p_best_scores[i]:

p_best_scores[i] = score

p_best_positions[i] = particles[i]

if score < g_best_score:

g_best_score = score

g_best_position = particles[i]

for i in range(num_particles):

velocities[i] = (w * velocities[i] +

c1 * np.random.rand() * (p_best_positions[i] - particles[i]) +

c2 * np.random.rand() * (g_best_position - particles[i]))

particles[i] += velocities[i]

particles[i] = np.clip(particles[i], -10, 10)

convergence.append(g_best_score)

# Convergence plot____

plt.plot(convergence)

plt.xlabel('Iteration')

plt.ylabel('Best Score')

plt.title('PSO Convergence Plot')

plt.grid(True)

plt.show()

best_coeffs = g_best_position

print("Best Coefficients:")

print(best_coeffs)

# Input data is denormalised for final prediction____

denormalized_data = np.copy(experimental_data)

denormalized_data[:, 0] = experimental_data[:, 0]

denormalized_data[:, 1] = experimental_data[:, 1]

denormalized_data[:, 2] = experimental_data[:, 2]

# Final predicted values are calculated____

predicted_values = []

for data in denormalized_data:

X, Y, Z = data[:3]

X = normalize(X, X_min, X_max)

Y = normalize(Y, Y_min, Y_max)

Z = normalize(Z, Z_min, Z_max)

predicted_values.append(thermal_conductivity(X, Y, Z, best_coeffs))

# Error percentages are calculated____

error_percentages = [(exp - pred) / exp * 100 for (exp, pred) in zip(experimental_data[:, 3], predicted_values)]

print("Error Percentages:")

print(error_percentages)

print("Predicted Values:")

print(predicted_values)
import matplotlib.pyplot as plt

# Scatter plot of Experimental vs. Predicted Thermal Conductivity

plt.figure(figsize=(8, 6))

plt.scatter(experimental_data[:, 3], predicted_values, color='blue', marker='o', label='Predicted vs Experimental')

plt.plot([min(experimental_data[:, 3]), max(experimental_data[:, 3])], [min(experimental_data[:, 3]), max(experimental_data[:, 3])], color='red', linestyle='--', label='Ideal Fit (y=x)')

plt.xlabel("Experimental Thermal Conductivity")

plt.ylabel("Predicted Thermal Conductivity")

plt.title("PSO Experimental vs Predicted Thermal Conductivity")

plt.legend()

plt.grid(True)

plt.show()

DFO

import numpy as np

import matplotlib.pyplot as plt

#Data Normalisation

def normalize(data):

return (data - np.min(data, axis=0)) / (np.max(data, axis=0) - np.min(data, axis=0))

#Polynomial model

def thermal_conductivity(X, Y, Z, coeffs):

return (coeffs[0] + coeffs[1] * X + coeffs[2] * Y + coeffs[3] * Z +

coeffs[4] * X**2 + coeffs[5] * Y**2 + coeffs[6] * Z**2 +

coeffs[7] * X * Y + coeffs[8] * X * Z + coeffs[9] * Y * Z)

#Experimental data

experimental_data = np.array([

[6, 150, 3, 0.769],

[2, 150, 1, 0.735],

[4, 150, 2, 0.782],

[2, 75, 2, 0.780],

[6, 225, 2, 0.788],

[4, 225, 1, 0.779],

[4, 225, 3, 0.780],

[4, 150, 2, 0.789],

[4, 75, 1, 0.720],

[4, 75, 3, 0.788],

[2, 150, 3, 0.788],

[6, 150, 1, 0.761],

[4, 150, 2, 0.786],

[6, 75, 2, 0.777],

[2, 225, 2, 0.768],

[5, 180, 2, 0.775] #Additional Data Point (not in original experimental data)

])

#Input data normalisation

X = normalize(experimental_data[:, :3])

Y = experimental_data[:, 3]

#Obj. fn definition

def objective_function(coeffs, X, Y, alpha=0.01):

predicted = thermal_conductivity(X[:, 0], X[:, 1], X[:, 2], coeffs)

mse = np.mean((Y - predicted) ** 2)

regularization = alpha * np.sum(coeffs ** 2) # L2 regularization

return mse + regularization

#DFO parameters

swarm_size = 50

dimensions = 10

max_iterations = 2000

initial_inertia_weight = 0.9

final_inertia_weight = 0.4

c = 1.0

s = 1.5

#Pos and Vel initialisation

positions = np.random.rand(swarm_size, dimensions)

velocities = np.zeros((swarm_size, dimensions))

personal_best_positions = np.copy(positions)

personal_best_scores = np.full(swarm_size, np.inf)

global_best_score = np.inf

global_best_coeffs = None

#Model Train

best_scores = []

for iteration in range(max_iterations):

inertia_weight = initial_inertia_weight - (initial_inertia_weight - final_inertia_weight) * (iteration / max_iterations)

for i in range(swarm_size):

fitness = objective_function(positions[i], X, Y)

if fitness < personal_best_scores[i]:

personal_best_positions[i] = positions[i]

personal_best_scores[i] = fitness

if fitness < global_best_score:

global_best_score = fitness

global_best_coeffs = positions[i]

for i in range(swarm_size):

for d in range(dimensions):

inertia = inertia_weight * velocities[i][d]

cognitive = c * np.random.rand() * (personal_best_positions[i][d] - positions[i][d])

social = s * np.random.rand() * (global_best_coeffs[d] - positions[i][d])

velocities[i][d] = inertia + cognitive + social

positions[i][d] += velocities[i][d]

best_scores.append(global_best_score)

if iteration % 100 == 0:

print(f"Iteration {iteration + 1}/{max_iterations}, Best Score: {global_best_score}")

print(f"Global Best Score: {global_best_score}")

print("Best Polynomial Coefficients:")

print(global_best_coeffs)

#Plot

plt.plot(best_scores)

plt.xlabel('Iteration')

plt.ylabel('Best Score')

plt.title('Convergence Plot')

plt.grid(True)

plt.xlim(0, 250) # Limit the x-axis to 250

plt.show()

predicted_values = thermal_conductivity(X[:, 0], X[:, 1], X[:, 2], global_best_coeffs)

predicted_values = np.maximum(predicted_values, 1e-6)

errors = ((predicted_values - Y) / Y) * 100

#Printed values

for i, (predicted, actual, error) in enumerate(zip(predicted_values, Y, errors)):

print(f"Data Point {i + 1}:")

print(f" Predicted Thermal Conductivity: {predicted:.6f}")

print(f" Actual Thermal Conductivity: {actual:.6f}")

print(f" Error Percentage: {error:.2f}%")

print()

import numpy as np

import matplotlib.pyplot as plt

# Assuming you have the variables `global_best_coeffs` and `experimental_data` from the main code

# Thermal conductivity model (same as in main code)

def thermal_conductivity(X, Y, Z, coeffs):

return (coeffs[0] + coeffs[1] * X + coeffs[2] * Y + coeffs[3] * Z +

coeffs[4] * X**2 + coeffs[5] * Y**2 + coeffs[6] * Z**2 +

coeffs[7] * X * Y + coeffs[8] * X * Z + coeffs[9] * Y * Z)

# Data normalization (same as in main code)

def normalize(data):

return (data - np.min(data, axis=0)) / (np.max(data, axis=0) - np.min(data, axis=0))

# Experimental data

experimental_data = np.array([

[6, 150, 3, 0.769],

[2, 150, 1, 0.735],

[4, 150, 2, 0.782],

[2, 75, 2, 0.780],

[6, 225, 2, 0.788],

[4, 225, 1, 0.779],

[4, 225, 3, 0.780],

[4, 150, 2, 0.789],

[4, 75, 1, 0.720],

[4, 75, 3, 0.788],

[2, 150, 3, 0.788],

[6, 150, 1, 0.761],

[4, 150, 2, 0.786],

[6, 75, 2, 0.777],

[2, 225, 2, 0.768],

[5, 180, 2, 0.775] # Additional Data Point

])

# Input data normalisation

X = normalize(experimental_data[:, :3])

Y = experimental_data[:, 3]

# Predicted values using the best coefficients (assuming you have these coefficients from your main code)

predicted_values = thermal_conductivity(X[:, 0], X[:, 1], X[:, 2], global_best_coeffs)

# Scatter plot for actual vs predicted values

plt.figure(figsize=(8, 6))

plt.scatter(Y, predicted_values, color='blue', marker='o', label='Predicted vs Experimental')

plt.plot([min(Y), max(Y)], [min(Y), max(Y)], color='red', linestyle='--', label='Ideal Fit (y=x)')

plt.xlabel("Experimental Thermal Conductivity")

plt.ylabel("Predicted Thermal Conductivity")

plt.title("DFO Experimental vs Predicted Thermal Conductivity")

plt.legend()

plt.grid(True)

plt.show()

# Print predicted values and errors

errors = ((predicted_values - Y) / Y) * 100

for i, (predicted, actual, error) in enumerate(zip(predicted_values, Y, errors)):

print(f"Data Point {i + 1}:")

print(f" Predicted Thermal Conductivity: {predicted:.6f}")

print(f" Actual Thermal Conductivity: {actual:.6f}")

print(f" Error Percentage: {error:.2f}%")

print()

CSA
import numpy as np

import matplotlib.pyplot as plt

np.random.seed(42)

#CSA Definition

def cuckoo_search(func, bounds, n_eggs=15, pa=0.25, alpha=0.01, max_iter=2000):

def levy_flight(Lambda):

sigma = (np.math.gamma(1 + Lambda) * np.sin(np.pi * Lambda / 2) /

(np.math.gamma((1 + Lambda) / 2) * Lambda * 2**((Lambda - 1) / 2))**(1 / Lambda))

u = np.random.randn(len(bounds)) * sigma

v = np.random.randn(len(bounds))

step = u / abs(v)**(1 / Lambda)

return step

#Generating initial eggs

eggs = np.random.rand(n_eggs, len(bounds))

for i in range(len(bounds)):

eggs[:, i] = eggs[:, i] * (bounds[i][1] - bounds[i][0]) + bounds[i][0]

best_egg = eggs[0]

best_fitness = func(best_egg)

fitness_history = []

for _ in range(max_iter):

for j in range(n_eggs):

step_size = alpha * levy_flight(1.5)

new_egg = eggs[j] + step_size * (eggs[j] - best_egg)

for i in range(len(bounds)):

new_egg[i] = np.clip(new_egg[i], bounds[i][0], bounds[i][1])

new_fitness = func(new_egg)

if new_fitness < func(eggs[j]):

eggs[j] = new_egg

if new_fitness < best_fitness:

best_egg = new_egg

best_fitness = new_fitness

fitness_history.append(best_fitness)

num_abandoned = int(pa * n_eggs)

for i in range(num_abandoned):

k = np.random.randint(0, n_eggs)

eggs[k] = np.random.rand(len(bounds))

for j in range(len(bounds)):

eggs[k, j] = eggs[k, j] * (bounds[j][1] - bounds[j][0]) + bounds[j][0]

return best_egg, best_fitness, fitness_history

#Defining the polynomial model for thermal conductivity

def thermal_conductivity(X, Y, Z, coeffs):

return (coeffs[0] + coeffs[1] * X + coeffs[2] * Y + coeffs[3] * Z +

coeffs[4] * X**2 + coeffs[5] * Y**2 + coeffs[6] * Z**2 +

coeffs[7] * X * Y + coeffs[8] * X * Z + coeffs[9] * Y * Z)

#Exp. data

experimental_data = np.array([

[6, 150, 3, 0.769],

[2, 150, 1, 0.735],

[4, 150, 2, 0.782],

[2, 75, 2, 0.780],

[6, 225, 2, 0.788],

[4, 225, 1, 0.779],

[4, 225, 3, 0.780],

[4, 150, 2, 0.789],

[4, 75, 1, 0.720],

[4, 75, 3, 0.788],

[2, 150, 3, 0.788],

[6, 150, 1, 0.761],

[4, 150, 2, 0.786],

[6, 75, 2, 0.777],

[2, 225, 2, 0.768],

[5, 180, 2, 0.775]

])

#Extract features and target values

X = experimental_data[:, 0]

Y = experimental_data[:, 1]

Z = experimental_data[:, 2]

T = experimental_data[:, 3]

#Normalize the input data

min_values = np.min(experimental_data[:, :3], axis=0)

max_values = np.max(experimental_data[:, :3], axis=0)

X_normalized = (X - min_values[0]) / (max_values[0] - min_values[0])

Y_normalized = (Y - min_values[1]) / (max_values[1] - min_values[1])

Z_normalized = (Z - min_values[2]) / (max_values[2] - min_values[2])

#Normalize the target data

T_min = np.min(T)

T_max = np.max(T)

T_normalized = (T - T_min) / (T_max - T_min)

#Define the objective function

def objective_function(coeffs):

predictions = thermal_conductivity(X_normalized, Y_normalized, Z_normalized, coeffs)

error = np.mean((predictions - T_normalized) ** 2)

return error

#Set bounds for coefficients

bounds = [(-1, 1)] * 10

#CSA Search

best_coeffs, best_fitness, fitness_history = cuckoo_search(objective_function, bounds)

#Calculate predictions and error percentages

predictions_normalized = thermal_conductivity(X_normalized, Y_normalized, Z_normalized, best_coeffs)

predictions = predictions_normalized * (T_max - T_min) + T_min

#error percentages

for i in range(len(T)):

error_percentage = ((predictions[i] - T[i]) / T[i]) * 100

print(f"Data Point {i+1}:")

print(f" Predicted Thermal Conductivity: {predictions[i]:.6f}")

print(f" Actual Thermal Conductivity: {T[i]:.6f}")

print(f" Error Percentage: {error_percentage:.2f}%\n")

print("Best Coefficients:", best_coeffs)

print("Best Fitness:", best_fitness)

plt.figure(figsize=(10, 6))

plt.plot(fitness_history[:500], color='blue')

plt.title("Cuckoo Search Convergence graph (till 500 iterations)")

plt.xlabel("Iteration")

plt.ylabel("Fitness (MSE)")

plt.grid(True)

plt.show()

# Scatter plot of Actual vs Predicted Thermal Conductivity

plt.figure(figsize=(8, 6))

plt.scatter(T, predictions, color='blue', label='Predicted vs Actual')

plt.plot([min(T), max(T)], [min(T), max(T)], color='red', linestyle='--', label='Ideal Fit')

plt.title("CSA Scatter Plot: Actual vs Predicted Thermal Conductivity")

plt.xlabel("Actual Thermal Conductivity")

plt.ylabel("Predicted Thermal Conductivity")

plt.legend()

plt.grid(True)

plt.show()

Code for showing convergence plot of all the three algorithms on a single graph:

import matplotlib.pyplot as plt

# Plotting all three algorithms' convergence with markers for each iteration

plt.figure(figsize=(10, 6))

# PSO Algorithm with markers

plt.plot(convergence, color='red', marker='o', markersize=4, linestyle='-', label='PSO ') #marker='o'

# DFO Optimization with markers

plt.plot(best_scores, color='green', marker='s', markersize=4, linestyle='-', label='DFO ') #marker='s'

# CSA Algorithm with markers

plt.plot(fitness_history, color='blue', marker='^', markersize=4, linestyle='-', label='CSA ') #marker = '^'

# Labels and title

plt.xlabel("Iteration")

plt.ylabel("Best Score / Fitness")

plt.xlim(0,1000)

plt.ylim(0,1.2)

plt.title("Convergence Plot of PSO, DFO, and CSA Algorithms (First 1000 Iterations)")

plt.legend() # Display the legend for color labels

plt.grid(True)

plt.show()
